# Drivers of Mobile Health Acceptance and Use From the Patient Perspective: Survey Study and Quantitative Model Development

**DOI:** 10.2196/17588

**Published:** 2020-07-09

**Authors:** Tânia Salgado, Jorge Tavares, Tiago Oliveira

**Affiliations:** 1 NOVA Information Management School Universidade Nova de Lisboa Lisbon Portugal

**Keywords:** digital health, mHealth, UTAUT2, health management, patient empowerment

## Abstract

**Background:**

Mobile health (mHealth) has potential to play a significant role in realizing a reversal of the current paradigm in health care toward a more patient-centric and more collaborative system to improve the outcomes obtained along with the quality and sustainability of health care systems.

**Objective:**

The aim of this study was to explore and understand individual mHealth acceptance drivers between two groups of users: those with chronic health conditions and those without.

**Methods:**

The extended unified theory of acceptance and usage of technology (UTAUT2) was enhanced with a new health-related framework: behavior intention to recommend and new mediation effects. We applied partial least squares (PLS) causal modeling to test the research model.

**Results:**

We obtained 322 valid responses through an online questionnaire. The drivers of behavior intention with statistical significance were performance expectancy (β=.29, *P*<.001), habit (β=.39, *P*<.001), and personal empowerment (β=.18, *P*=.01). The precursors of use behavior were habit (β= .47, *P*<.001) and personal empowerment (β=.17, *P*=.01). Behavior intention to recommend was significantly influenced by behavior intention (β=.58, *P*<.001) and personal empowerment (β=.26, *P*<.001). The model explained 66% of the total variance in behavior intention, 54% of the variance in use behavior, and 70% of the variance in behavior intention to recommend.

**Conclusions:**

Our study demonstrates a significant role of personal empowerment, as a second-order construct, in the mHealth acceptance context. The presence of a chronic health condition predicates an impact on acceptance of this technology.

## Introduction

### Context

The focus of health policymakers worldwide is to change health care models from disease treatment to disease prevention, necessitating a shift to more patient-centric and more collaborative actions, and information technology is one of the paths highlighted to best achieve this goal [1-5]. In this study, we focused on the use of mobile health (mHealth), specifically on smartphones, as a health management platform. In Portugal, smartphone penetration was reported to reach up to 74.9% of the population in December 2017 [6]. The field of mHealth is in a state of rapid expansion, from a global rate of 36% use in 2016 to 46% in 2018, and nearly half (48%) of all health care consumers were using mobile/tablet apps compared to only 16% reported in 2014 [7].

The main limitations of previously published research on mHealth include underpowered pilot data [8] in specific groups of patients or with a particular app [1], or a focus on only health care professionals [9,10]. In this study, we developed a new research model to explore and better understand individual mHealth acceptance drivers, mainly to determine how unique drivers related to health care such as patient empowerment can influence the adoption of mHealth among health care consumers with and without a chronic condition.

### Theoretical Background

mHealth is a subset of the larger field of electronic health (eHealth), which was originally defined under the term “unwired e-med” [11]. Many definitions have arisen since then, and in the present work, we follow the World Health Organization definition of mHealth as the use of mobile devices such as mobile phones, patient monitoring devices, personal digital assistants, and wireless devices for medical and public health practice [12].

In the last two decades, mainly after the emergence of mobile phones followed by smartphones, several researchers have been studying electronic and mobile technologies as possible solutions to address health care challenges. Relevant studies performed in the most recent years are summarized in Table 1.

**Table 1:** Electronic health (eHealth) adoption models.

Theory	Dependent variable	Findings	Reference
TAM^a^, HBM^b^ and TPB^c^	Health consumers’ behavior intention of using HIT^d^	-PT^e^, PU^f^, and PEOU^g^ significantly affected health consumers’ attitude and behavior intention. -Health consumers’ health status, health belief and concerns, subjective norm, HIT characteristics, and HIT self-efficacy had a substantial indirect impact on attitude and behavioral intention through the mediators of PT, PU, and PEOU.	[13]
TAM and TPB	Internet use for health purposes	- Positive influence of perceived health risk and health consciousness on health-related internet use - Perceived health risk positively affects health-related internet use - Health consciousness has a significant positive effect on health-related internet use - Health consciousness contributes to health behavior adoption	[14]
TAM2, Dual-factor model, HBM	Patient’s acceptance of smartphone health technology for chronic disease management	- PU of the app was positively influenced by the perceived health threat, relationship with doctor, and PEOU, but negatively affected by resistance to change - Usage experience and self-efficacy positively influenced patients’ PEOU - Behavior intention was influenced by enablers of PU and perceived health threat, an inhibitor of resistance to change - Intention of use had a significant weak relationship with actual use	[1]
UTAUT2^h^	EHR^i^ portals	- Understanding of the adoption of EHR portals is improved through the use of consumer adoption-specific constructs	[15]
Uses and gratification theory	eHealth usage by sociodemographic factors	- Female gender is a consistent predictor of eHealth usage - Age is primarily influential for health-information seeking	[16]
TAM, UTAUT2	Consumer acceptance of wearable self-tracking devices	- Positive influence of trust, perceived esthetics, personal innovativeness, perceived support of health, perceived support of fitness, and perceived support of well-being on consumer acceptance of wearables	[17]
DMISSM^j^	Evaluation of trust, security beliefs, and privacy of HIT as determinants of health care outcomes	- Increased privacy concerns reduce the frequency of patient access to health records use, positive attitudes toward HIT, and perceptions of patient care quality - Belief in the effectiveness of information security increases the frequency of patient access to health records and a positive attitude toward HIT - Trust in health information had a positive association with attitudes toward HIT and perceived patient care quality	[18]
Personal empowerment	Internet use behavior as a source of information	- Health information seeking is analyzed under three perspectives: professional logic, consumer logic, and community logic	[19]
DIT^k^	Factors impacting patient acceptance and use of consumer eHealth innovations	Health care providers need to consider and address patient characteristics, their social system, and preferences on communication channels, as well as the attributes of the innovation to guarantee its success.	[20]

^a^TAM: technology acceptance model.

^b^HBM: health belief model.

^c^TPB: theory of planned behavior.

^d^HIT: health information technology.

^e^PT: perceived threat.

^f^PU: perceived usefulness.

^g^PEOU: perceived ease of use.

^h^UTAUT2: extended unified theory of acceptance and usage of technology.

^i^EHR: electronic health record.

^j^DMISSM: DeLone and McLean information systems success model.

^k^DIT: diffusion of innovation theory.

The technology acceptance model (TAM) is the most widely used research model in this field, which is commonly combined with other models or with extensions to help explain behavior intention or use behavior. An example of this approach is a study that combined the health belief model (HBM) and TAM to explore the influence of perceived health risk and health consciousness on health-related internet use [14]. In addition, Kim and Park [13] combined the TAM, HBM, and theory of planned behavior to describe health consumers’ behavior intention of using health information technologies. Dou et al [1] used a combination of TAM2, the dual-factor model, and HBM to study patients’ acceptance of smartphone health technology for chronic disease management, among others.

### Extended Unified Theory of Acceptance and Usage of Technology

The extended version of the unified theory of acceptance and usage of technology (UTAUT), UTAUT2, is a tailor-made model of acceptance and use of technology. UTAUT2 was adapted from the UTAUT to a more consumer-centered context, with the main differences including the introduction of three new constructs, hedonic motivation, price value, and habit, with moderators of age, gender, and experience. Hedonic motivation and price value explain behavior intention, and habit explains both behavior intention and use behavior. Compared to UTAUT, the extensions proposed in UTAUT2 resulted in a substantial improvement in the variance explained in behavior intention (56% to 74%) and technology use (40% to 52%) [21].

### Research Model and Hypotheses

UTAUT2 has already shown good results when applied to the health care context, with the addition of specific health-related constructs [22-24]. Concerning specific health care adoption models, the aim is to achieve an *R^2^* value of at least 50% [21,25], except when the model deals with constructs related to sensitive topics such as confidentiality or patient behavior in which an *R^2^* of 20% for the critical dependent variables can be regarded as acceptable [25,26]. If the model uses an existing adoption theory, extended by new constructs or relationships related to health care, these should be able to produce statistically significant results [21,25].

**Figure 1:**
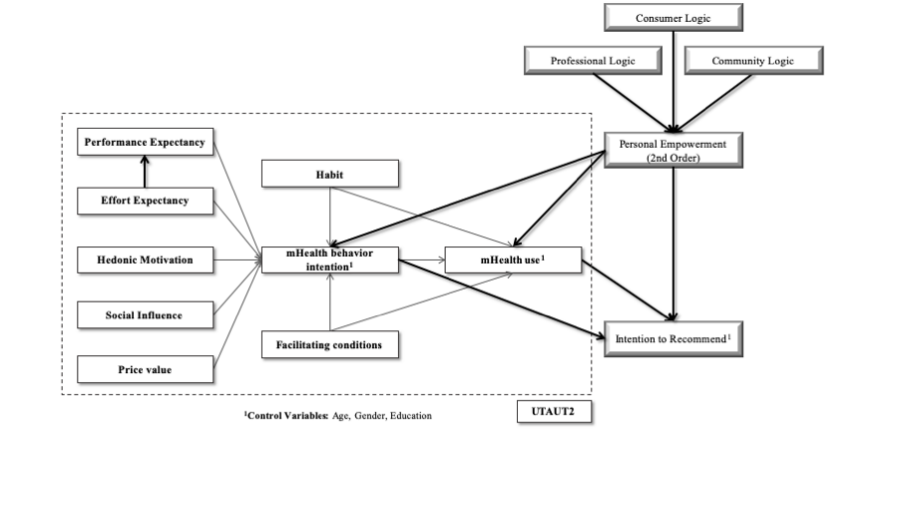
Research model. Original relationships from unified theory of acceptance and usage of technology (UTAUT2) are in black; new relationships from the extended model are in grey.

The need for change in health care models related to the scarceness of human resources and to improve health outcomes emphasizes the need to empower patients to manage their own health [27], and mHealth is considered as one of the privileged means to achieve this goal [28]. Accordingly, and due to the lack of studies using a personal/patient empowerment construct (ie, a construct associated with other drivers of information technology adoption), we here propose a model that combines UTAUT2 and personal empowerment as a second-order construct following the work of Lemire [19], which is outlined in Figure 1.

The hypotheses of this study were developed using the rationale of Venkatesh et al [21] with some adjustments. First, we dropped experience, as our model will be applied at a single time point. Age, gender, and education were used as control variables. In addition, the presence of chronic health conditions was added considering our intention to analyze the results between groups with and without these conditions We established the following research hypotheses based on the following constructs.

#### Performance Expectancy

Performance expectancy is defined as the consumers’ projections of benefits provided by the use of technology and is a good predictor of behavior intention, including in the eHealth context [22].

H1(a): Performance expectancy will positively influence behavior intention.

H1(b): Performance expectancy will positively influence behavior intention in the chronic condition group, and there will be a significantly greater effect when compared to that of the healthy group.

#### Effort Expectancy

Effort expectancy is associated with how easy and simple it seems to be to use a particular technology. Earlier research on the usability of eHealth showed conflicting results, which was likely related to differences in study populations; nevertheless, in the present work, we have followed the UTAUT2 rationale. Assuming that consumers who perceive mHealth to be more useful and easier to use would have a higher intention to use it [13], we also add a new mediation effect hypothesis:

H2(a): Effort expectancy will positively influence behavior intention.

H2(b): Effort expectancy will mediate the influence of performance expectancy on behavior intention.

#### Hedonic Motivation

Hedonic motivation is defined by the fun or pleasure derived from using technology [21]. A previous study using health and fitness apps demonstrated that hedonic motivation drives behavior intention [29].

H3: Hedonic motivation will positively influence behavior intention.

#### Social Influence

Social influence is the degree of consumers’ perception that people who are significant to them believe they should use technology. This effect has also been verified in the case of eHealth [15].

H4: Social influence will positively influence behavior intention.

#### Facilitating Conditions

The construct of facilitating conditions is defined as consumer awareness of the existing support to use technology. Earlier studies also suggested that if patients with a chronic condition have the needed resources and support available, they are more likely to use eHealth technologies [30].

H5(a): Facilitating conditions will positively influence behavior intention.

H5(b): Facilitating conditions will positively influence use behavior.

H5(c): Facilitating conditions will positively influence behavior intention in the chronic conditions group, and there will be a significant increase when compared to the healthy group.

H5(d): Facilitating conditions will positively influence use behavior in the chronic conditions group, and there will be a significant increase when compared to the healthy group.

#### Price Value

Price value refers to the advantages obtained from a technology considering the costs of using it [21]. The use of mHealth technologies not only offers an easy way to reach health services (eg, scheduling appointments, examination results) but also provides a privileged way to continually monitor patients’ parameters, allowing for better follow up with less traveling to health care institutions.

H6: Price value will positively influence behavior intention.

#### Habit

Habit refers to the automatic nature of a behavior response resulting from learning. As a result of prior experiences, habit has been demonstrated as a good predictor of the adoption of different technologies [21]. Therefore, we have tested this aspect for mHealth adoption.

H7(a): Habit will positively influence behavior intention.

H7(b): Habit will positively influence use behavior.

#### Behavior Intention

According to previous research on eHealth, the act of using eHealth tools is preceded by the behavior intention to use them [24]; therefore, we applied this concept to the mHealth context.

H8(a): Behavior intention will positively influence use behavior.

Considering the previous hypotheses, and according to earlier research about technology adoption in the health care information technology context indicating that intention to use a technology strongly influences its recommendation to others [22,31], we developed the following hypotheses for testing in the mHealth context.

H8(b): Behavior intention will positively influence the intention to recommend mHealth technologies to others.

H9: Use behavior will positively influence the intention to recommend mHealth technologies to others.

#### Personal Empowerment

Personal empowerment is the process and outcome through which individuals gain self-confidence and self-efficacy to actively participate in their own health care and ultimately exercise power over decision making concerning their treatment [19]. Therefore, the behavior intention and use of mHealth technologies will be positively influenced by personal empowerment, since it allows for a more active role in health management. We used personal empowerment as a second-order construct. This construct is based on three different forms of logic: professional logic, the process of empowerment in which individuals acquire expert knowledge and put it into practice so that they can act effectively in their personal health; consumer logic, the process of personal affirmation to make decisions based on one’s own judgment and resources; and community logic, the dynamics of inclusion in action and social change initiatives developed from a sense of community and participation. Following the rationale of H9, we also hypothesized that behavior intention to recommend technology is influenced by personal empowerment in the same way.

H10(a): Personal empowerment will positively influence behavior intention.

H10(b): Personal empowerment will positively influence mHealth use.

H10(c): Personal empowerment will positively influence behavior intention to use.

H10(d): Personal empowerment will positively influence behavior intention in the chronic conditions group, which will be significantly higher when compared to that of the healthy group.

H10(e): Personal empowerment will positively influence mHealth use in the chronic conditions group, which will be significantly higher when compared to that of the healthy group.

H10(f): Personal empowerment will positively influence behavior intention in the chronic conditions group, which will be significantly higher when compared to that of the healthy group.

## Methods

### Measurement

All of the measurement items for each of the constructs described above were adapted from Venkatesh et al [21], Tavares and Oliveira [22,24], and Lemire et al [19] with minor modifications to adapt to the mHealth context. The items are described in detail in Multimedia Appendix 1. The questionnaire was developed in English and then translated to Portuguese, which was validated by two translators fluent in both languages. To guarantee that the questionnaire did not lose its original meaning, a back-translation was made to English by a third translator with no previous knowledge of the original questionnaire, which was then compared with the original [32]. The scale items were measured on a 7-point Likert scale from “strongly disagree” (1) to “strongly agree” (7). An exception was made for the use behavior construct, which was measured on a different scale ranging from “never” (1) to “whenever I need” (7) for most of the items following the study of Tavares and Oliveira [24]. Some sociodemographic questions were also added to characterize the study sample. Age was measured in years and gender was coded as a dummy variable (0 or 1), with women represented by 0. The presence of a chronic health condition was also coded as a dummy variable (0 or 1), with its absence represented by 0. Each respondent’s education level was assessed by 5 different layers (1, middle school or lower; 2, high school; 3, bachelor degree or postgraduate; 4, master degree; 5, doctorate). The questionnaire started with a brief introduction explaining the mHealth concept (Multimedia Appendix 1) to ensure that all respondents had prior knowledge and contact with mHealth technologies.

### Data Collection

To validate the questions and the scales of the survey, a pilot survey was conducted, from which we received 40 responses, assuring that all of the items were reliable and valid. These data were not included in the main survey. According to previous literature on health information technologies, the users of mHealth are mostly younger and have higher education levels [16]; therefore, one of the targets selected for our sample was educational institutions. In addition, considering the goal of analyzing the impact of having chronic health conditions on the use of mHealth technologies, we requested the collaboration of a set of national patients’ associations to diffuse our study among their associates and allow us to reach a sample of individuals specifically with these characteristics. By the end of January 2019, an email with a collaboration request and the survey hyperlink was sent to 6 educational institutions in Lisbon and 30 national chronic patients’ associations. The request included the study purpose and a statement that anonymity and confidentiality of the information collected were assured, and that by following the hyperlink, they would authorize the use of the data for academic purposes. After this first approach, we received 118 responses. Reminders were sent at 3 and 6 weeks after sending the first email to improve the response rate. Following the reminders, we obtained a total of 322 respondents, including 209 from educational institutions and 113 from the patients’ associations. We verified the common method bias through Harman’s one-factor test [33] that attests that the total variance for every single factor is always less than 50%. Using Lindell and Whitney’s [34] approach, we found a maximum shared variance of 6.5% with other variables after adding a theoretically irrelevant marker variable in the research model, which can be considered low [35]. Therefore, using two independent approaches, we demonstrated that common method bias should not be an issue.

In our total sample, approximately 71% (229/322) of the respondents were women, the average age was 40 years, and 77% were university graduates or higher, which is in line with the literature [20,36]. The patients with chronic health conditions were older on average than the healthy group, and there were also differences in education level between the two groups. The basic characteristics of our sample are shown in Table 2 in more detail.

**Table 2:** Sample characteristics.

Characteristic			Total (N=322)		Chronic conditions group (n=124)		Healthy group (n=198)
Age (years), mean (SD)			40.03 (13.70)		47.66 (14.30)		35.26 (10.89)
**Gender, n (%)**							
	Male	93 (28.9)		34 (27.4)		59 (29.8)	
	Female	229 (71.1)		90 (72.6)		139 (70.2)	
**Education level, n (%)**							
	Middle school or lower	11 (3.4)		8 (6.5)		3 (1.5)	
	High school	61 (18.9)		37 (29.8)		24 (12.1)	
	Bachelor or postgraduate	163 (50.6)		54 (43.6)		109 (55.1)	
	Master	72 (22.4)		17 (13.7)		55 (27.8)	
	Doctorate	15 (4.7)		8 (6.5)		7 (3.5)	

### Data Analysis

We used a structural equation model (SEM)-partial least square (PLS) approach to analyze the data obtained. Smart PLS v3.28 software [37] was used to analyze the relationships defined by the model. PLS is a causal modeling approach and a powerful multivariate analysis technique that enables analysis of the complexity of the model and to test the validity of the theory using empirical models [15,38]. The rationale for choosing this approach was the high model complexity (many constructs and many indicators), the incorporation of formative measured constructs as part of the structural model, and the fact that the PLS-SEM method is oriented to explain the variance of the research model and to detect statistically significant constructs [25,39].

## Results

### Measurement Model 

Since we had both reflective and formative indicators, we applied different measures to assess the reliability and validity of the measurement model. For reflective indicators, we initially evaluated the internal consistency reliability through Cronbach alpha and composite reliability. Table 3 shows that both measures were above .70, demonstrating internal consistency [25]. Validity was examined by the convergent validity and discriminant validity metrics. Convergent validity is assured when each item has outer loadings above 0.70 and when each construct’s average variance extracted is 0.50 or higher. As shown in Table 3, both of these criteria were met. Discriminant validity represents the extent to which a construct is empirically distinct from other constructs [40], which was evaluated with three methods: analysis of crossloadings (the outer loading of an indicator should be higher than all its crossloadings), as verified in Table 3; the Fornell and Larcker criterion, which states that the square root of the average variance extracted for each construct should be greater than its higher correlation with others constructs, as confirmed in Table 4; and the Heterotrait-monotrait ratio, which is an estimate of the true correlation between constructs and should be below 0.90 [25], as confirmed in Multimedia Appendix 2.

**Table 3:** Total sample loadings and crossloadings.

Constructs			PE^a^		EE^b^		SI^c^		HT^d^		FC^e^		HM^f^		PV^g^		PEM^h^		PEM-PL^i^		PEM-CL^j^		PEM-CCL^k^		BI^l^		BIR^m^		CA^n^			CR^o^			AVE^p^	
**PE**																														.90			0.94			0.84
	PE1	*0.87* ^q^		0.37		0.44		0.52		0.31		0.40		0.23		0.52		0.51		0.50		0.40		0.56		0.55										
	PE2	*0.93*		0.51		0.39		0.54		0.47		0.49		0.39		0.54		0.55		0.52		0.39		0.61		0.58										
	PE3	*0.94*		0.48		0.38		0.52		0.41		0.42		0.37		0.55		0.59		0.52		0.39		0.63		0.59										
**EE**																														.93			0.95			0.82
	EE1	0.43		*0.92*		0.12		0.37		0.62		0.41		0.37		0.31		0.32		0.32		0.21		0.32		0.30										
	EE2	0.51		*0.93*		0.27		0.50		0.66		0.51		0.43		0.39		0.39		0.37		0.30		0.42		0.38										
	EE3	0.41		*0.87*		0.22		0.42		0.61		0.48		0.45		0.39		0.39		0.34		0.32		0.34		0.35										
	EE4	0.44		*0.91*		0.13		0.41		0.64		0.43		0.41		0.34		0.36		0.33		0.24		0.38		0.30										
**SI**																														.97			0.98			0.94
	SI1	0.43		0.21		*0.97*		0.58		0.29		0.40		0.27		0.55		0.52		0.47		0.48		0.47		0.48										
	SI2	0.43		0.18		*0.97*		0.59		0.26		0.44		0.28		0.54		0.54		0.44		0.48		0.51		0.52										
	SI3	0.40		0.21		*0.96*		0.59		0.28		0.43		0.31		0.55		0.56		0.45		0.47		0.51		0.52										
**HT **																														.87			0.91			0.73
	HT1	0.52		0.46		0.53		*0.92*		0.45		0.59		0.41		0.53		0.54		0.46		0.43		0.64		0.56										
	HT2	0.31		0.21		0.49		*0.74*		0.23		0.51		0.28		0.47		0.42		0.40		0.45		0.44		0.42										
	HT3	0.61		0.39		0.55		*0.83*		0.40		0.57		0.44		0.62		0.64		0.53		0.49		0.71		0.68										
	HT4	0.50		0.51		0.51		*0.92*		0.51		0.60		0.45		0.57		0.58		0.50		0.47		0.68		0.61										
**FC**																														.86			0.91			0.71
	FC1	0.34		0.59		0.22		0.38		*0.86*		0.40		0.49		0.40		0.41		0.39		0.29		0.38		0.37										
	FC2	0.40		0.70		0.25		0.44		*0.88*		0.45		0.50		0.38		0.39		0.37		0.27		0.43		0.36										
	FC3	0.44		0.64		0.25		0.45		*0.91*		0.52		0.57		0.43		0.44		0.42		0.32		0.43		0.36										
	FC4	0.29		0.39		0.26		0.31		*0.71*		0.41		0.52		0.40		0.43		0.33		0.33		0.33		0.30										
**HM**																														.90			0.94			0.84
	HM1	0.47		0.50		0.40		0.62		0.52		*0.95*		0.47		0.57		0.53		0.48		0.52		0.54		0.52										
	HM2	0.52		0.53		0.43		0.70		0.54		*0.93*		0.53		0.60		0.59		0.50		0.53		0.61		0.55										
	HM3	0.29		0.31		0.36		0.46		0.36		*0.87*		0.40		0.45		0.40		0.35		0.45		0.42		0.39										
**PV **																														.92			0.95			0.87
	PV1	0.31		0.40		0.21		0.36		0.57		0.42		*0.92*		0.47		0.44		0.45		0.38		0.39		0.37										
	PV2	0.35		0.43		0.28		0.46		0.58		0.47		*0.96*		0.50		0.49		0.47		0.40		0.50		0.47										
	PV3	0.35		0.45		0.33		0.48		0.57		0.54		*0.92*		0.55		0.53		0.51		0.45		0.49		0.49										
**PEM-PL**																														.92			0.94			0.76
	PEM-PL1	0.52		0.39		0.56		0.59		0.45		0.51		0.52		0.82		*0.91*		0.67		0.63		0.57		0.61										
	PEM-PL2	0.54		0.37		0.51		0.62		0.45		0.52		0.51		0.82		*0.91*		0.68		0.63		0.59		0.61										
	PEM-PL3	0.54		0.33		0.51		0.54		0.40		0.50		0.44		0.85		*0.93*		0.68		0.67		0.56		0.60										
	PEM-PL4	0.58		0.40		0.47		0.58		0.46		0.51		0.43		0.81		*0.89*		0.66		0.63		0.61		0.62										
	PEM-PL5	0.46		0.27		0.39		0.46		0.38		0.42		0.38		0.67		*0.72*		0.58		0.52		0.47		0.47										
**PEM-CL**																														.90			0.93			0.77
	PEM-CL1	0.56		0.39		0.45		0.54		0.43		0.45		0.43		0.80		0.73		*0.85*		0.61		0.57		0.59										
	PEM-CL2	0.47		0.28		0.37		0.43		0.35		0.38		0.45		0.74		0.57		*0.89*		0.60		0.47		0.49										
	PEM-CL3	0.50		0.34		0.45		0.49		0.40		0.46		0.49		0.81		0.66		*0.92*		0.67		0.54		0.58										
	PEM-CL4	0.43		0.31		0.38		0.49		0.39		0.45		0.44		0.80		0.67		*0.85*		0.69		0.51		0.55										
**PEM-CCL**																														.93			0.95			0.78
	PEM-CCL1	0.37		0.24		0.43		0.47		0.29		0.48		0.36		0.78		0.60		0.63		*0.87*		0.49		0.51										
	PEM-CCL2	0.37		0.24		0.38		0.43		0.27		0.45		0.38		0.78		0.60		0.63		*0.90*		0.45		0.49										
	PEM-CCL3	0.38		0.31		0.45		0.51		0.38		0.53		0.41		0.81		0.64		0.63		*0.91*		0.56		0.55										
	PEM-CCL4	0.46		0.30		0.48		0.53		0.34		0.52		0.43		0.85		0.71		0.67		*0.90*		0.60		0.59										
	PEM-CCL5	0.31		0.19		0.43		0.41		0.28		0.44		0.35		0.75		0.57		0.64		*0.83*		0.45		0.47										
**BI**																														.95			0.97			0.90
	BI1	0.65		0.38		0.44		0.66		0.42		0.51		0.46		0.63		0.62		0.56		0.53		*0.95*		0.76										
	BI2	0.58		0.34		0.53		0.71		0.40		0.56		0.43		0.63		0.59		0.54		0.56		*0.94*		0.77										
	BI3	0.66		0.44		0.50		0.71		0.50		0.59		0.52		0.66		0.63		0.60		0.56		*0.97*		0.78										
**BIR **																														.93			0.95			0.82
	BIR1	0.59		0.33		0.53		0.65		0.36		0.53		0.48		0.67		0.63		0.61		0.58		0.77		*0.93*										
	BIR2	0.53		0.36		0.32		0.50		0.37		0.40		0.44		0.54		0.54		0.50		0.41		0.64		*0.84*										
	BIR3	0.58		0.33		0.51		0.65		0.38		0.52		0.42		0.65		0.62		0.59		0.57		0.77		*0.95*										
	BIR4	0.57		0.32		0.51		0.61		0.38		0.51		0.40		0.66		0.63		0.58		0.58		0.73		*0.90*										

^a^PE: performance expectation.

^b^EE: effort expectancy.

^c^SI: social influence.

^d^HT: habit.

^e^FC: facilitation conditions.

^f^HM: hedonic motivation.

^g^PV: price value.

^h^PEM: personal empowerment (second order).

^i^PEM-PL: personal empowerment-professional logic.

^j^PEM-CL: personal empowerment-consumer logic.

^k^PEM-CCL: personal empowerment-community logic.

^l^BI: behavior intention.

^m^BIR: behavior intention to recommend.

^n^CA: Cronbach alpha.

^o^CR: composite reliability.

^p^AVE: average variance extracted.

^q^Numbers in italics indicate loadings of the indicators for their own constructs.

**Table 4:** Total sample descriptive statistics, correlations, and square root of average variance extracted.

Variable	Mean (SD)	PE^a^	EE^b^	SI^c^	HT^d^	FC^e^	HM^f^	PV^g^	PEM^h^	PEM-PL^i^	PEM-CL^j^	PEM-CCL^k^	BI^l^	BIR^m^	Age	Education	Gender	PCHC^n^
PE	4.90 (1.63)	0.91^o^																
EE	4.90 (1.63)	0.50	0.91^o^															
SI	5.41 (1.34)	0.44	0.21	0.97^o^														
HT	5.18 (1.55)	0.58	0.47	0.60	0.85^o^													
FC	4.49 (1.58)	0.44	0.70	0.28	0.47	0.84^o^												
HM	3.84 (1.89)	0.48	0.51	0.44	0.67	0.53	0.91^o^											
PV	5.23 (1.36)	0.37	0.46	0.30	0.47	0.62	0.51	0.93^o^										
PEM	4.47 (1.54)	0.59	0.40	0.56	0.64	0.48	0.60	0.54	0.79^o^									
PEM-PL	4.21 (1.57)	0.61	0.40	0.56	0.64	0.49	0.56	0.53	0.91	0.87^o^								
PEM-CL	4.44 (1.54)	0.56	0.38	0.47	0.56	0.45	0.49	0.51	0.90	0.75	0.88^o^							
PEM-CCL	4.75 (1.46)	0.43	0.29	0.49	0.53	0.35	0.55	0.44	0.90	0.71	0.73	0.88^o^						
BI	4.34 (1.40)	0.66	0.41	0.51	0.73	0.47	0.58	0.50	0.67	0.64	0.60	0.58	0.95^o^					
BIR	4.04 (1.63)	0.63	0.37	0.52	0.67	0.41	0.54	0.48	0.70	0.67	0.63	0.60	0.81	0.90^o^				
Age	40.04 (13.6)	–0.08	–0.29	0.24	0.05	–0.10	0.03	–0.11	0.04	0.09	–0.05	0.04	–0.01	0.00	1.00			
Education	N/A^p^	0.06	0.20	–0.10	–0.03	0.25	0.08	0.11	0.02	0.03	0.06	–0.03	0.05	0.08	–0.12	1.00		
Gender	N/A	0.20	0.03	0.15	0.12	0.10	0.13	0.06	0.22	0.22	0.21	0.16	0.17	0.18	0.13	–0.02	1.00	
PCHC	N/A	–0.04	–0.11	0.15	0.11	–0.02	0.11	–0.06	0.04	0.11	–0.08	0.04	0.03	0.05	0.44	–0.20	–0.03	1.00

^a^PE: performance expectation.

^b^EE: effort expectancy.

^c^SI: social influence.

^d^HT: habit.

^e^FC: facilitation conditions.

^f^HM: hedonic motivation.

^g^PV: price value.

^h^PEM: personal empowerment (second order).

^i^PEM-PL: personal empowerment-professional logic.

^j^PEM-CL: personal empowerment-consumer logic.

^k^PEM-CCL: personal empowerment-community logic.

^l^BI: behavior intention.

^m^BIR: behavior intention to recommend.

^n^PCHC: presence of chronic health condition.

^o^Square root of average variance extracted.

^p^N/A: not applicable.

Use behavior is formed by 10 formative indicators, and its assessment involves specific quality criteria. No collinearity issues were detected in the total model with a variance inflation factor (VIF) below 5 for all indicators. Besides not all the indicators’ weights complying with the criteria of being statistically significant, their outer loadings were all higher than 0.5, with some exceptions. Nevertheless, since all of the outer loadings were statistically significant, we retained all of the indicators in the model (see Table 5).

**Table 5:** Formative indicators for quality criteria.

Indicator	VIF^a^	Loading	Weight	Loading *P* value	Weight *P* value
UB^b^f1: What is your actual frequency of use of mHealth^c^ to collect biometric data for medical follow-up?	2.10	0.74	0.21	<.001	.007
UBf2: What is your actual frequency of use of mHealth to collect biometric data related to well-being (fitness apps)?	1.68	0.84	0.45	<.001	<.001
UBf3: What is your actual frequency of use of mHealth to access a patient portal (eg, manage appointments, results of clinical analysis, application for online prescription)?	1.94	0.77	0.34	<.001	<.001
UBf4: What is your actual frequency of use of mHealth to monitor therapeutic compliance/adhesion (prescribed drugs/medicine intake follow up)?	2.01	0.53	–0.06	<.001	.43
UBf5: What is your actual frequency of use of mHealth for scientific observational studies (eg, medicine, app, or innovative treatment trial)?	1.86	0.52	0.07	<.001	.45
UBf6: What is your actual frequency of use of mHealth for health information research?	1.80	0.64	0.11	<.001	.21
UBf7: What is your actual frequency of use of mHealth for clinical screening and counselling?	2.04	0.64	0.17	<.001	.04
UBf8: What is your actual frequency of use of mHealth for making remote medical consultations/appointments?	1.90	0.42	0.08	<.001	.34
UBf9: What is your actual frequency of use of mHealth to request home medical consultation?	1.64	0.31	–0.14	<.001	.05
UBf10: What is your actual frequency of use of mHealth to participate in peer support groups or online communities of patients?	1.81	0.46	0.07	<.001	.48

^a^VIF: variance inflation factor.

^b^UB: use behavior.

^c^mHealth: mobile health.

Personal empowerment is designed as a reflective formative–type higher-order construct [25,41]. We assessed its multicollinearity according to the VIF, which indicated no collinearity issues as the VIF varied from 2.44 to 2.76 (ie, <5), and all of the weights were statistically significant and positive (Table 6).

**Table 6:** Measurement model evaluation for the higher-order formative constructs personal empowerment.

Constructs	VIF^a^	Weight	*P* value
Personal Empowerment - Community Logic	2.44	0.38	<.001
Personal Empowerment - Consumer Logic	2.76	0.32	<.001
Personal Empowerment - Professional Logic	2.61	0.41	<.001

^a^VIF: variance inflation factor.

These same assessments were also applied separately to the two groups under analysis (with and without a chronic health condition), as shown in Multimedia Appendix 3. Considering the results, we concluded that all of the constructs were suitable to test the conceptual model.

### Structural Model

Before assessing the structural model, we first tested the multicollinearity of all constructs based on the VIF. All VIF values were below the threshold of 5, ranging from 1.00 to 2.73, indicating the absence of multicollinearity among the variables.

The structural model path significance levels were estimated using bootstrap resampling with 5000 iterations to achieve the maximum possible consistency in the results. The *R^2^* value was used to assess the structural model. The total model explained 66% of the variance in behavior intention, 54% of the variance in use behavior, and 70% of the variance in behavior intention to recommend.

We performed a PLS multigroup analysis to analyze the two groups in our sample. However, the results obtained were not globally statistically significant. Nevertheless, analysis of the two groups independently demonstrated some significant differences with comparison. Behavior intention explained a higher percentage of the variance in the model for the patients with chronic health conditions than for the healthy group (74% vs 65%), whereas higher variance for use behavior and behavioral intention to recommend was found for the healthy group than for the chronic conditions group (63% vs 51% and 75% vs 62%, respectively). Table 7 summarizes the detailed structural model results (*R^2^*, path coefficients significance, and significance between groups).

**Figure 2:**
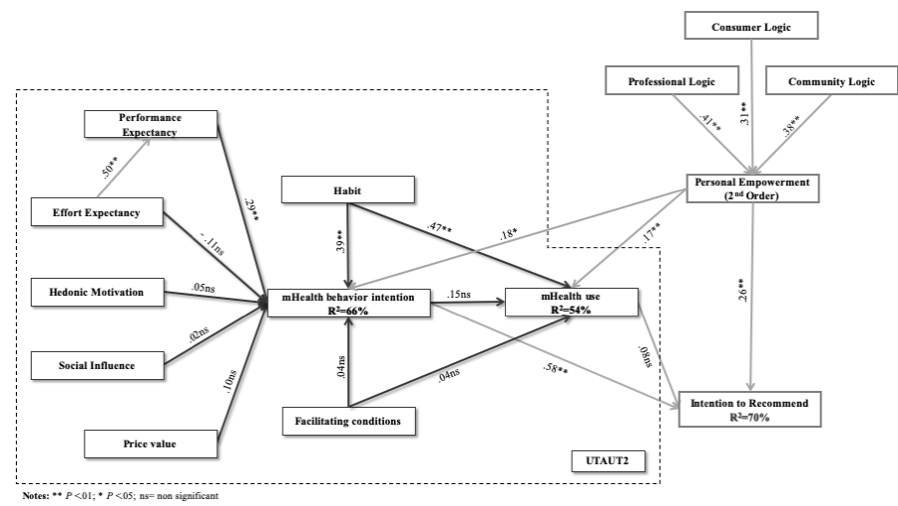
Results of the structural model for the total sample. UTAUT: unified theory of acceptance and usage of technology; mHealth: mobile health.

**Figure 3:**
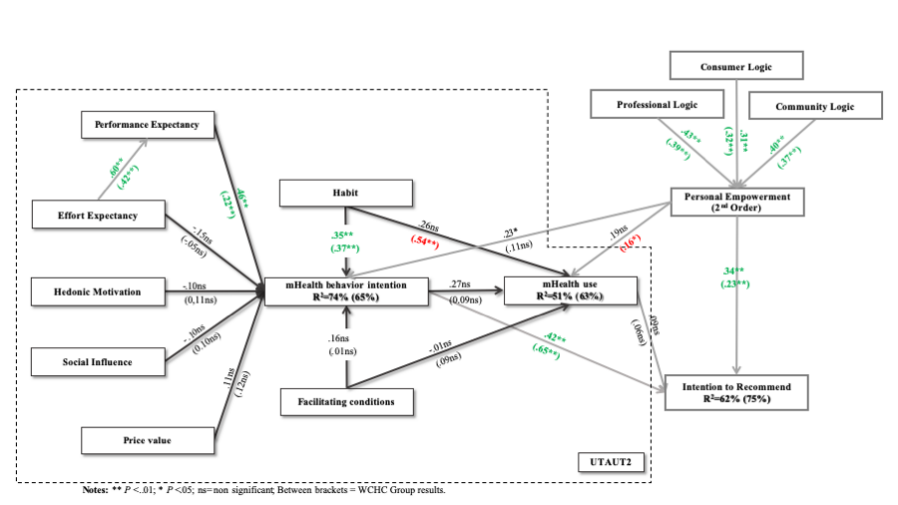
Structural model results for the chronic health conditions patients (CHCP) group and the without chronic health conditions (WCHC) group (in parentheses). Statistically significant relationships observed for both groups are presented in green. Significant relationships only in the WCHC group are presented in red. Results with no significant relationship in either group are presented in black with ns.

Figure 2 and Figure 3 show the structural model results of the total model and for each group, respectively.

**Table 7:** Structural model results.

Dependent/independent variables		β				*t* value						*R*^*2*^ (%)					
		Total	CHP^a^	WCHC^b^	CHCP – WCHC		Total	CHCP	WCHC	CHCP – WCHC			Total		CHCP		WCHC
**BI^c^**											66			74		65	
	PE^d^	.29^**^	.46^**^	.22^**^	.19		5.13^**^	5.20^**^	3.13^**^	1.68							
	EE^e^	–.11	–.15	–.05	.15		1.76	1.43	0.71	1.07							
	SI^f^	.02	–.10	.11	.04		0.38	1.79	1.36	0.43							
	HT^g^	.39^**^	.35^**^	.37^**^	.12		5.68^**^	3.23^**^	4.20^**^	0.84							
	FC^h^	.04	.16	.00	.07		0.56	1.42	0.06	0.46							
	HM^i^	.05	–.10	.10	.16		0.87	1.01	1.51	1.25							
	PV^j^	.10	.11	.12	.11		1.74	1.26	1.69	1.04							
	PEM^k^	.18^*^	.23^*^	.11	.02		2.55^*^	2.47^*^	1.10	0.14							
	Age	–.04	–.03	–.03	.00		1.06	0.54	0.70	0.00							
	Gender	.02	.00	.03	.04		0.64	0.09	0.79	0.55							
	Education	.04	.02	.02	.00		1.06	0.27	0.36	0.02							
**UB^1^**											54			51		63	
	HT	.47^**^	.26	.54^**^	.23		6.16^**^	1.67	6.09^**^	1.40							
	FC	.04	–.01	.09	.14		0.68	0.13	1.44	1.17							
	PEM	.17^**^	.19	.16^*^	.01		2.62^**^	1.44	2.19	0.04							
	BI	.15	.27	.09	.23		1.83	1.76	0.96	1.38							
	Age	–.04	–.20^*^	.07	.26		0.72	2.44^*^	1.26	2.87^**^							
	Gender	.02	.06	.00	.07		0.47	0.81	0.06	0.74							
	Education	.10^*^	.10	.05	.05		2.33^*^	1.12	0.92	0.56							
**BIR^m^**											70			62		75	
	PEM	.26^**^	.34^**^	.23^**^	.09		4.67^**^	3.49^**^	3.56^**^	0.85							
	BI	.58^**^	.42^**^	.65^**^	.11		9.17^**^	4.19^**^	8.26^**^	0.82							
	UB	.08	.09	.06	.15		1.56	0.43	0.99	1.43							
	Age	–.01	.00	–.03	.03		0.35	0.02	0.83	0.40							
	Gender	.01	.01	.00	.01		0.37	0.17	0.06	0.10							
	Education	.04	.10	.03	.07		1.38	1.73	0.78	1.08							

^a^CHCP: patients with chronic health conditions.

^b^WCHC: without chronic health conditions.

^c^BI: behavior intention.

^d^PE: performance expectation.

^e^EE: effort expectancy.

^f^SI: social influence.

^g^HT: habit.

^h^FC: facilitation conditions.

^i^HM: hedonic motivation.

^j^PV: price value.

^k^PEM: personal empowerment (second order).

^l^UB: use behavior.

^m^BIR: behavior intention to recommend.

** *P*<.01, *P<.05; df (bootstrap)=4999.

## Discussion

### Principal Findings

The results of our study support development of the UTAUT2 model with constructs that are more specific to the health domain. Most of the constructs of UTAUT2 did not show statistical significance in the sample analyzed in this study. In contrast, an important role of personal empowerment was revealed for the intention to use, use behavior, and behavior intention to recommend mHealth. Further, the addition of behavior intention to recommend mHealth had a substantial contribution to explaining the mHealth phenomenon with a high *R^2^* value. The mediation effect of effort expectancy on performance expectancy influenced behavior intention, demonstrating a relevant role to effort expectancy in the understanding of mHealth. However, when focusing on the group of patients with chronic health conditions, the results were not as meaningful as expected, disclosing significance of the independent variables only on behavior intention. These results lead to an important concern that patients with chronic conditions, who would more likely receive benefits from the use of mobile technology to manage their health condition, not only still do not use it as often as they could (mean use behavior 3.32, SD 0.84, range 1-7) but also their intention to use is not a predecessor of use.

### Theoretical Implications

According to the results summarized in Table 8, performance expectancy and habit were predictors of behavior intention in the total model and across the two groups analyzed (with and without chronic conditions) with a positive statistically significant impact, supporting H1(a) and 7(a). Previous studies addressing the eHealth context reported similar results [15].

The impact of performance expectancy on behavioral intention in the chronic conditions group was highly significant (β=.46, *P*<.001), confirming our rationale that the expectancy of the benefits obtained with the use of mHealth technology to manage health strongly influences the intention of using it for patients with chronic conditions. Nevertheless, the hypothesized difference between groups related to performance expectancy was not verified, thereby not supporting H1(b).

The predictor effect of effort expectancy has shown contradictory results in previous studies [42,43]. In our sample, the influence of effort expectancy on behavioral intention was not statistically significant, thereby not supporting H2(a). However, the proposed mediation effect of effort expectancy for the influence of perceived effort on behavioral intention was statistically significant, revealing a full mediation effect, since only the indirect effect of effort expectancy was significant [25], which supports H2(b).

The personal empowerment construct that we included in the model showed its importance to the study with a positive statistically significant impact on behavior intention in the total model and in the chronic conditions group. For use behavior, this construct revealed a statistically significant positive impact in the full sample and in the healthy group, and also had a statistically significant positive impact in all three analyses when related to behavioral intention to recommend, thereby corroborating H10(a), H10(b), and H10(c). Nevertheless, the expected difference between the chronic conditions and healthy groups was not confirmed, thereby not supporting H10(d), H10(e), and H10(f). This may also explain why hedonic motivation and social influence were not found to have significant effects (H3 and H4 not supported). Since personal empowerment, as a second-order construct, has a personal/consumer and a community logic, inclusion of these concepts can better capture a model considering the health context.

Our results do not support the influence of facilitation conditions on behavioral intention or use behavior, thereby not supporting H5(a) and H5(b), and the difference expected between groups was also not verified, thereby not supporting H5(c) and H5(d). This element suggests that the individuals in our sample consider that the resources or knowledge to use mHealth are not an issue, which is likely related to the current natural increasing access to a mobile phone and mobile internet. According to the 2017 ANACOM mobile services report [6], the number of mobile broadband users in Portugal reached 7.2 million, representing an increase of 9.5% from the prior year.

Price value also did not show a significant effect in our sample, thereby not supporting H6. The absence of influence could be related to the Portuguese national health service universal coverage concept, with health care services tending to be free, but also with the fact that eHealth/mHealth models such as teleconsultation are not yet very common in Portugal. Another reason may be that benefits other than the price of the technology are not being perceived by end users.

**Table 8:** Summary of findings regarding the hypotheses.

Hypothesis	Path	β	*t* value	Result
1 (a)	PE^a^ to BI^b^	.29	5.13^**^	Supported
1 (b)	(PE_CHCP_^c^ to BI_CHCP_) - (PE_WCHC_^d^ to BI_WCHC_)	.19	1.68	Not supported
2(a)	EE^e^ to BI	–.11	1.76	Not supported
2(b)	EE to PE to BI	.27	4.47^**^	Supported
3	HM^f^ to BI	.05	0.87	Not supported
4	SI^g^ to BI	.02	0.38	Not supported
5 (a)	FC^h^ to BI	.04	0.56	Not supported
5(b)	FC to UB^i^	.04	0.68	Not supported
5(c)	(FC_CHCP_ to BI_CHCP_) - (FC_WCHC_ to BI_WCHC_)	.07	0.46	Not supported
5(d)	(FC_CHCP_ to UB_CHCP_) - (FC_WCHC_ to UB_WCHC_)	.14	1.17	Not supported
6	PV^j^ to BI	.10	1.74	Not supported
7(a)	HT^k^ to BI	.39	5.68^**^	Supported
7(b)	HT to UB	.47	6.16^**^	Supported
8(a)	BI to UB	.15	1.83	Not supported
8(b)	BI to BIR^l^	.58	9.17^**^	Supported
9	UB to BIR	.08	1.56	Not supported
10(a)	PEM^m^ to BI	.18	2.55^*^	Supported
10(b)	PEM to UB	.17	2.62^**^	Supported
10(c)	PEM to BIR	.26	4.67^**^	Supported
10(d)	(PEM_CHCP_ to BI_CHCP_) - (PEM_WCHC_ to BI_WCHC_)	.02	0.14	Not supported
10(e)	(PEM_CHCP_ to UB_CHCP_) - (PEM_WCHC_ to UB_WCHC_)	.01	0.04	Not supported
10(f)	(PEM_CHCP_ to BIR_CHCP_) - (PEM_WCHC_ to BIR_WCHC_)	.09	0.85	Not supported

^a^PE: performance expectation.

^b^BI: behavior intention.

^c^CHCP: patients with chronic conditions.

^d^WCHC: without chronic conditions.

^e^EE: effort expectancy.

^f^HM: hedonic motivation.

^g^SI: social influence.

^h^FC: facilitation conditions.

^i^UB: use behavior.

^j^PV: price value.

^k^HT: habit.

^l^BIR: behavior intention to recommend.

^m^PEM: personal empowerment.

** *P*<.01, *P<.05; df (bootstrap)=4999.

In contrast to the UTAUT2 rationale, our results do not attest to the influence of behavioral intention on use behavior, thereby not supporting H8(a). The same conclusion was reached in a study by Lim et al [44] on acceptance of using mobile phones to seek health information by women in Singapore, which raises the concern that behavior intentions are not translated in actual use, but only on the intention to recommend, thereby supporting H8(b). This aspect is even more relevant when we consider only the patients with chronic conditions in which none of the independent variables showed statistical significance on use behavior.

Our control variables were globally not statistically significant, with the exception of a significant impact of age in the chronic conditions group. This outcome shows that younger individuals tend to use mHealth more than older individuals [45]. In addition, in the total sample, higher education levels were associated with a higher level of use of this technology, which is in accordance with expectations and in line with previous research [16]. Gender was not statistically significant for any group or dependent variable.

Overall, our results reinforce the importance of performance expectancy and habit as drivers of technology acceptance, which is aligned with previous studies, and specifically in mHealth acceptance. Moreover, we found a significant effect of personal empowerment, demonstrating the beneficial effect of adding this aspect to UTAUT2 when analyzing the acceptance of mobile technology in a health management context.

### Health Policy Implications

The results of our study show that personal empowerment is a key driver of mHealth. This suggests that governments worldwide should focus more on patient-centric policies, with more direct communication to the patients to promote their empowerment as this will drive the adoption of mHealth. Personal empowerment has been studied in many contexts and with different levels of significance [46]. Our results show how consumers truly value this aspect from the eHealth context. The anticipated scarcity of human health care resources in the near future emphasizes how critical it is to involve the patient in the management of their own health, leading to a change of the health care paradigm to a more predictive, personalized, preventive, and participatory approach [47], which has been promoted by emerging technologies. Owing to its proximity, accessibility, and increasing diffusion, mHealth is one of the privileged paths to foster this transformation, increasing not only the interactivity between patients and health care providers but also the engagement of individuals, underlining their active and determinant role as key actors in their health management cycle to help toward democratizing health care [48]. Currently, mHealth users are diverse, being not only patients but also general consumers who aim to be more informed and diligent in every aspect of their life, particularly with respect to health-related issues. Therefore, this aspect must be considered as a primary driver to the integration of this technology as part of health care systems.

Effort expectancy did not show significant predictive power as a direct influencer of behavioral intention, which is in opposition to the results of a UTAUT2 study and other previous studies [43]. Nevertheless, effort expectancy had a strong mediator effect on performance expectancy, indicating the need to provide more information to the public about mHealth. Such clarification refers to its applications and ease of use, and to reinforce its role as a pivotal tool to ease the interaction and proximity between patients and health care providers in such a way that promotes its integration in health care management.

Our research shows that mHealth technologies still have low usage in Portugal, as demonstrated by the use behavior descriptive statistics. Therefore, targeting early adopters and its continuance of use can be one of the main strategies adopted to increase the diffusion of mHealth technologies by fostering word-of-mouth recommendation. This was attested by the significant role of habit and the good results of behavioral intention to recommend. This outcome shows that governments and health care institutions should realize that the current users of mHealth also play a role in the diffusion of mHealth tools. Therefore, this aspect should be considered as part of the promotion strategy that governments and health care institutions implement to increase the adoption of mHealth tools.

Experts, clinical/managerial staff, and health care providers should also be aware that behavior intention is only a proxy for measuring technology acceptance [49]. More specifically, a positive behavioral intention does not always translate into the actual use of technology, as demonstrated in the present study. The ease to operate a technology, health and eHealth literacy level, belief in the importance of having an active role in one’s own health management, and even the relationship with health care providers [50] also have to be considered.

Another important topic to take in account is that the highest risk group (ie, patients that are older and have chronic health conditions [51]) demonstrated a lower use of mHealth. The majority of the features showed higher usage rates in the younger group (see Table 9) as expected; however, we would like to highlight that older people (aged ≥55 years) mentioned that they use mHealth more than the younger group to request home medical appointments (UBf9) and to obtain information about scientific studies and new treatments (UBf5). Clearly, these two aspects show that older people with chronic diseases are concerned about their health. This is intuitive as a chronic condition gets worse with age [51], and patients will therefore seek information about treatments with increasing age, which also impacts their mobility so that remote appointments are a good solution [51]. From a standpoint of health policy, by addressing these topics to the older population in a more effective manner, such as by promoting and providing support for the use of remote medical consultations, the problem of lack of mobility for the older population with chronic conditions can be solved while also allowing for more effective management of health care resources in the future that will become increasingly scarce to manage the growing increase of chronic diseases related with aging [3]. For example, with the COVID-19 outbreak, it is even more critical to make sure that older people with chronic health conditions remain at home in isolation [52,53]. Nevertheless, this population still needs to be in contact with health care providers; therefore, communication and remote home medical appointments are excellent options to keep these high-risk people safe via mHealth.

**Table 9:** Median use behavior (UB) formative indicators in patients with chronic conditions according to age.

UB formative indicators	Younger patients (<55 years; n=44), median	Older patients (≥55 years; n=80), median
UBf1: What is your actual frequency of use of mHealth to collect biometric data for medical follow-up?	4.00	3.00
UBf2: What is your actual frequency of use of mHealth to collect biometric data related to well-being (fitness apps)?	4.00	3.00
UBf3: What is your actual frequency of use of mHealth to access a patient portal (eg, manage appointments, results of clinical analysis, application for online prescription)?	6.00	5.00
UBf4: What is your actual frequency of use of mHealth to monitor therapeutic compliance/adhesion (prescribed drugs/medicine intake follow up)?	4.00	3.00
UBf5: What is your actual frequency of use of mHealth for scientific observational studies (eg, medicine, app, or innovative treatment trial)?	2.00	3.00
UBf6: What is your actual frequency of use of mHealth for health information research?	5.00	5.00
UBf7: What is your actual frequency of use of mHealth for clinical screening and counselling?	3.50	3.50
UBf8: What is your actual frequency of use of mHealth for making remote medical consultations/appointments?	1.00	1.00
UBf9: What is your actual frequency of use of mHealth to request home medical consultation?	1.00	1.50
UBf10: What is your actual frequency of use of mHealth to participate in peer support groups or online communities of patients?	2.00	2.00

### Limitations and Future Research

One of the limitations of this study was the use of a convenience sampling approach, which was limited to educational institutions and patients’ associations, thereby restraining the extrapolation of the results for the general population. The cross-sectional design of the study, which did not allow to capture changes over time, and the geographical circumscription are additional limitations. Namely, mHealth is still at an early use stage in Portugal, and the national health system, besides being in a transition process, is still very “paternalistic,” meaning there is a highly dependent relationship between health care professionals and patients, which follows a model of care that is strongly focused on institutions such as hospitals [54]. Therefore, future studies may benefit from a cross-country comparison, ideally including countries at different stages of mHealth adoption so as to explore better paths to increase the use of mHealth. In this context, it would be very interesting to develop a longitudinal study and add to the research experience information, at least in a self-reported manner, and the continuance of use constructs.

Additionally, regarding the extension of UTAUT2, application of the moderation effects of the original model, such as experience considering that habit had a significant impact on the dependent variables, or even the exploration of new effects, could improve the explanatory power of the model. Our research goal focused on understanding whether having a chronic condition/disability would influence the acceptance drivers of mHealth. However, we only collected this information as a binary coded variable, without details on the type or severity. Therefore, future researchers could develop this perspective with more detail. Another important topic in future studies would be to increase the sample size, particularly in the group of patients with chronic diseases, because age (as a control variable) had a significant effect in use for this group. Patients with chronic diseases have a higher risk at older age [3]. A relevant sample size (eg, ≥100 individuals per age group) [25] could allow for conducting a subgroup analysis between older and younger patients with chronic conditions.

### Conclusion

mHealth technologies are suggested as one of the privileged means to address emerging problems in health care, including the need to improve access to health services, regardless of time and place [27]. Governments worldwide are concerned about the increased prevalence of chronic diseases and age-related diseases with regard to the potentially insufficient human health care resources available in the future to deal with this new situation. Our study showed that promoting the engagement of patients in their own self-care via mHealth can be a viable solution for this problem [27]. In addition, mHealth can present a viable solution for high-risk patients such as older patients with chronic diseases that need to be in isolation to protect them from infectious diseases like COVID-19 [52,53]. These patients can be reached by health care providers remotely using mHealth. The primary goal of this study, using a novel theoretical model, was to explore and understand individual mHealth acceptance drivers, and to further explore if having a chronic health condition influences these drivers. The tested model incorporates an extension of UTAUT2 with personal empowerment and behavior intention to recommend constructs, and established a new mediation effect between perceived effort and behavior intention through effort expectancy.

The study was conducted in Portugal with a sample of 322 individuals recruited from educational institutions and patients’ associations, and the model explained 66% of the variance of behavior intention, 54% of use behavior, and 70% of behavioral intention to recommend. Performance expectancy and habit emerged as predictors of behavior intention in the total model and across the two groups analyzed (those with and without chronic health conditions). Effort expectancy had a significant effect on the influence of perceived effort to behavior intention, revealing the impact of this construct. In the chronic conditions group, the percentage of behavior intention variance explained by the model was higher than that obtained in the healthy group (74% vs 65%), whereas the percentages for use behavior and behavior intention to recommend were higher in the healthy group than in the chronic conditions group (63% vs 51% and 75% vs 62%, respectively). The personal empowerment construct had a significant effect on behavior intention in the total model and for the chronic conditions group. Personal empowerment had a positive impact on use behavior in the total sample and the healthy group, and had a significant effect on behavior intention to recommend for the total sample as well as in each of the two groups.

Overall, our findings show that by using constructs that are specifically health-related, namely personal empowerment as a second-order construct, we achieved a model that could offer a better explanation of mHealth acceptance drivers. With this study, we advance the perspective of technology acceptance at the individual/patient level, thereby reinforcing the existing knowledge and highlighting the need for further research to develop more evidence-based theories in this field.

## Appendix

Multimedia Appendix 1 Questionnaire items.

Multimedia Appendix 2 Total sample heterotrait-monotrait ratio (HTMT).

Multimedia Appendix 3 Group analysis tables.

## Abbreviations

eHealth: electronic health
HBM: health belief model
mHealth: mobile health
PLS: partial least squares
SEM: structural equation model
TAM: technology acceptance model
UTAUT: Unified theory of acceptance and usage of technology
UTAUT2: extended unified theory of acceptance and usage of technology
VIF: variance inflation factor
