# Supplementary material for: Drivers of Mobile Health Acceptance and Use From the Patient Perspective: Survey Study and Quantitative Model Development
Source: JMIR Mhealth Uhealth. 2020 Jul 9;8(7):e17588. doi: 10.2196/17588 (PMC7380904; doi:10.2196/17588)
Supplement: Multimedia Appendix 1 [file mhealth_v8i7e17588_app1.docx]

**MULTIMEDIA APPENDIX 1**

**Introduction**

According to the World Health Organization (WHO), mobile health (M-health) consists in the use of mobile devices for medical and health practices. When we mention "m-Health" in this questionnaire, we refer specifically to the use of the smartphone to health management issues. This technology is currently widely used, namely by mobile applications (apps) use to assess for instance the level of physical activity and/or monitoring physiological parameters (heart rate, glucose level, blood pressure, sleep...), to health information research, to access electronic health portals (to appointments scheduling, consultation of test results...), among many others.

| **Construct** | | **Code** | **Items** | **Reference** |
| --- | --- | --- | --- | --- |
| **Performance Exp ectancy** | | PE1 | Using m-health technologies will support critical aspects of my healthcare. | [24] |
|  |  | PE2 | Using m-health technologies will enhance my effectiveness in managing my healthcare. |  |
|  |  | PE3 | Overall, m-health technologies will be useful in managing my healthcare. |  |
| **Effort Expectancy** | | EE1 | Learning how to use m-health technologies is easy for me. | [21] |
|  |  | EE2 | My interaction with m-health technologies is clear and understandable. |  |
|  |  | EE3 | I find m-health technologies easy to use. |  |
|  |  | EE4 | It is easy for me to become skilful at using m-health technologies. |  |
| **Social Influence** | | SI1 | People who are important to me think that I should use m-health technologies. | [21] |
|  |  | SI2 | People who influence my behaviour think that I should use m-health technologies. |  |
|  |  | SI3 | People whose opinions that I value prefer that I use m-health technologies. |  |
| **Habit** | | HT1 | The use of m-health technologies has become a habit for me. | [21] |
|  |  | HT2 | I am addicted to using m-health technologies. |  |
|  |  | HT3 | I must use m-health technologies. |  |
|  |  | HT4 | Using m-health technologies has become natural to me. |  |
| **Facilitating**  **Conditions** | | FC1 | I have the resources necessary to use m-health technologies. | [21] |
|  |  | FC2 | I have the knowledge necessary to use m-health technologies. |  |
|  |  | FC3 | m-health technologies are compatible with other technologies I use. |  |
|  |  | FC4 | I can get help from others when I have difficulties using m-health technologies. |  |
| **Hedonic**  **Motivation** | | HM1 | Using m-health technologies is fun. | [21] |
|  |  | HM2 | Using m-health technologies is enjoyable. |  |
|  |  | HM3 | Using m-health technologies is very entertaining. |  |
| **Price value** | | PV1 | M-health technologies are reasonably priced. | [21] |
|  |  | PV2 | M-health technologies are a good value for the money. |  |
|  |  | PV3 | At the current price, m-health technologies provide a good value. |  |
| **Personal**  **Empowerment**  2^nd^ Order  Construct | Professional    Logic | PE-PL1 | The m-health technologies allow me to be better informed about how to follow the advice of the physician or professionals I consult. | [19] |
|  |  | PE-PL2 | The use of m-health technologies allows me to develop a better  understanding of my personal health giving me access to recognized expert knowledge |  |
|  |  | PE-PL3 | The m-health technologies help me feel better equipped for implementing the advice of the physician or health professionals I consult. |  |
|  |  | PE-PL4 | Because of what I discover using m-health technologies, I plan on playing a more active role in my health care by carefully following the advice of my physician or health professionals I consult. |  |
|  |  | PE-PL5 | I am determined that from now on, I will make decisions on my health without, however, going against the advice of the physician or the health professionals I have consulted. |  |
|  |  | PE-CCL5 | I am determined that from now on I will make decisions about my health by relying on the experiences and points of view of the people with whom I talk (on the Internet, at work, in my family, etc.) |  |

| **Construct** |  | **Code** | **Items** | **Reference** |
| --- | --- | --- | --- | --- |
|  | Cons  umer Logic | PE-CL1 | The use of m-health technologies allows me to become better informed so that I can make my own health-related choices/decisions. |  |
|  |  | PE-CL2 | The m-health technologies help me feel better equipped to make my own choices, without being limited to the advice of a physician or health professional, which I believe is the best approach. |  |
|  |  | PE-CL3 | The m-health technologies make me feel more confident about the choices I plan on making, on my own, between the various possible treatments and solutions. |  |
|  |  | PE-CL4 | Because of what I discover on the m-health technologies, I plan on playing a more active role in my healthcare by deciding which solutions I prefer, whether from mainstream medicine or alternative approaches. |  |
|  | Community Logic | PE-CCL1 | M-health technologies allow me to know more about the opinions of people who are in similar situations or who are active in support groups. |  |
|  |  | PE-CCL2 | M-health technologies allow me to better understand my personal health through online discussions or the opinions of people going through similar experiences. |  |
|  |  | PE-CCL3 | The m-health technologies help me feel better equipped to make positive changes to my situation through discussions and exchanges with others (in my family, at work, on the Internet, etc.) |  |
|  |  | PE-CCL4 | Because of what I discover using m-health technologies, I plan on playing a more active role in my health care by continuing to talk with the people in my life who could help me clarify my ideas. |  |
|  |  | PE-CCL5 | I am determined that from now on I will make decisions about my health by relying on the experiences and points of view of the people with whom I talk (on the Internet, at work, in my family, etc.) |  |
| **Behaviour Intention** |  | BI1 | I intend to continue using m-health technologies in the future. | [21] |
|  |  | BI2 | I will always try to use m-health technologies in my daily life. |  |
|  |  | BI3 | I plan to continue to use m-health technologies frequently. |  |
| **Use Behaviour** |  |  | What is your actual frequency of use of m-health to: | [22,24] |
|  |  | UB1 | Collect biometric data to medical follow-up (blood pressure, heart rate, blood sugar, etc. …) |  |
|  |  | UB2 | Collect biometric data related to wellbeing (fitness apps) |  |
|  |  | UB3 | Access to a patient portal (manage appointments, access to results of clinical analysis, application for online prescription, etc.) |  |
|  |  | UB4 | Monitor therapeutic compliance/adhesion (prescribed drugs/medicine intake follow up) |  |
|  |  | UB5 | Scientific observational study (e.g. medicine, app or innovative treatment trial) |  |
|  |  | UB6 | Health information research |  |
|  |  | UB7 | Clinical screening and counselling (use of digital health platforms with artificial intelligence algorithms to analyze symptoms, e.g. ADA health) |  |
|  |  | UB8 | Remote medical consultation/appointment (or with other healthcare professionals) |  |
|  |  | UB9 | Request home medical consultation |  |
|  |  | UB10 | Participate in peer support groups or online communities of patients |  |
| **Behaviour**  **Intention to**  **Recommend** |  | BIR1 | I will recommend my friends/family to use m-health technologies, if available. | [22] |
|  |  | BIR2 | If I have a good experience with m-health technologies, I will recommend my friends/family to use it. |  |
|  |  | BIR3 | If m-health technologies help me to better manage my health, I will recommend my friends/family. |  |
|  |  | BIR4 | I will recommend m-health technologies to my friends/family because it helps me to be more aware of my health status. |  |
