# Supplementary material for: Drivers of Mobile Health Acceptance and Use From the Patient Perspective: Survey Study and Quantitative Model Development
Source: JMIR Mhealth Uhealth. 2020 Jul 9;8(7):e17588. doi: 10.2196/17588 (PMC7380904; doi:10.2196/17588)
Supplement: Multimedia Appendix 2 [file mhealth_v8i7e17588_app2.pdf]

MULTIMEDIA APPENDIX 2

Table A.2.1 Total Sample Heterotrait-Monotrait Ratio (HTMT)

CONSTRUCTS	PE	EE	SI	HT	FC	HM	PV	PEM	PEM-PL	PEM-CL	PEM-CCL	BI	BIR		AGE	EDUCATION	GENDER	PCHC
**PE**																		
**EE**	0.54																	
**SI**	0.47	0.21																
**HT**	0.64	0.51	0.66															
**FC**	0.49	0.77	0.32	0.53														
**HM**	0.51	0.53	0.46	0.73	0.59													
**PV**	0.40	0.49	0.31	0.51	0.69	0.55												
**PEM**	0.63	0.42	0.59	0.70	0.53	0.63	0.58											
**PEM-PL**	0.66	0.44	0.59	0.71	0.56	0.61	0.57	0.97										
**PEM-CL**	0.62	0.41	0.50	0.62	0.51	0.54	0.56	0.97	0.82									
**PEM-CCL**	0.47	0.31	0.52	0.60	0.40	0.60	0.47	0.96	0.76	0.79								
**BI**	0.71	0.43	0.53	0.79	0.51	0.62	0.53	0.70	0.69	0.64	0.62							
**BIR**	0.69	0.40	0.55	0.74	0.46	0.58	0.51	0.74	0.72	0.69	0.64	0.86						
**AGE**	0.08	0.31	0.24	0.06	0.10	0.03	0.11	0.07	0.09	0.07	0.04	0.03	0.02					
**EDUCATION**	0.07	0.20	0.10	0.04	0.27	0.08	0.12	0.05	0.04	0.07	0.04	0.06	0.09		0.12			
**GENDER**	0.21	0.05	0.15	0.12	0.11	0.14	0.06	0.22	0.23	0.22	0.17	0.18	0.18		0.13	0.02		
**PCHC**	0.04	0.11	0.15	0.12	0.04	0.11	0.06	0.09	0.12	0.08	0.04	0.04	0.05		0.44	0.20	0.03	

Notes: PE: Performance Expectancy; EE: Effort Expectancy; SI: Social Influence; HT: Habit; FC: Facilitation Conditions; HM: Hedonic Motivation; PV: Price Value; PEM: Personal Empowerment (2nd order);PEM-PL: Personal Empowerment – Professional Logic; PEM-CL: Personal Empowerment- Consumer Logic; PEM-CCL: Personal Empowerment – Community Logic; BI: Behaviour Intention; Abuse Behaviour BIR: Behaviour Intention to Recommend; PCHC: Presence of Chronic Health Condition.
