# Supplementary material for: Drivers of Mobile Health Acceptance and Use From the Patient Perspective: Survey Study and Quantitative Model Development
Source: JMIR Mhealth Uhealth. 2020 Jul 9;8(7):e17588. doi: 10.2196/17588 (PMC7380904; doi:10.2196/17588)
Supplement: Multimedia Appendix 3 [file mhealth_v8i7e17588_app3.pdf]

# MULTIMEDIA APPENDIX 3

**Table A.3.1** CHC Patients Sample Loadings and cross-loadings

| Constructs | Item | PE | EE | SI | HT | FC | HM | PV | PEM | PEMPL | PEMCL | PEM-CCL | BI | BIR |
|---|---|---|---|---|---|---|---|---|---|---|---|---|---|---|
| **Performance Expectancy (PE)** | **PE1** | **0.85** | 0.26 | 0.52 | 0.47 | 0.26 | 0.34 | 0.21 | 0.51 | 0.53 | 0.52 | 0.38 | 0.50 | 0.51 |
| CA=.93 | **PE2** | **0.93** | 0.44 | 0.44 | 0.49 | 0.43 | 0.44 | 0.38 | 0.56 | 0.58 | 0.57 | 0.40 | 0.58 | 0.58 |
| CR=0.95 | **PE3** | **0.94** | 0.43 | 0.41 | 0.48 | 0.39 | 0.38 | 0.34 | 0.56 | 0.60 | 0.54 | 0.39 | 0.58 | 0.58 |
| AVE=0.78 | | | | | | | | | | | | | | |
| **Effort Expectancy (EE)** | **EE1** | 0.35 | **0.91** | 0.06 | 0.26 | 0.58 | 0.32 | 0.33 | 0.30 | 0.35 | 0.30 | 0.16 | 0.25 | 0.22 |
| CA=.92 | **EE2** | 0.45 | **0.93** | 0.21 | 0.43 | 0.62 | 0.46 | 0.36 | 0.40 | 0.43 | 0.40 | 0.28 | 0.38 | 0.33 |
| CR=0.94 | **EE3** | 0.34 | **0.85** | 0.11 | 0.35 | 0.53 | 0.39 | 0.38 | 0.39 | 0.43 | 0.35 | 0.30 | 0.28 | 0.29 |
| AVE=0.81 | **EE4** | 0.36 | **0.90** | 0.09 | 0.32 | 0.63 | 0.38 | 0.38 | 0.32 | 0.38 | 0.31 | 0.20 | 0.30 | 0.23 |
| **Social Influence (SI)** | **SI1** | 0.49 | 0.15 | **0.96** | 0.63 | 0.28 | 0.41 | 0.21 | 0.52 | 0.57 | 0.58 | 0.52 | 0.55 | 0.55 |
| CA=.95 | **SI2** | 0.48 | 0.11 | **0.98** | 0.64 | 0.25 | 0.48 | 0.25 | 0.56 | 0.59 | 0.54 | 0.56 | 0.61 | 0.61 |
| CR=0.97 | **SI3** | 0.46 | 0.15 | **0.97** | 0.66 | 0.31 | 0.47 | 0.29 | 0.53 | 0.59 | 0.55 | 0.53 | 0.60 | 0.60 |
| AVE=0.90 | | | | | | | | | | | | | | |
| **Habit (HT)** | HT1 | 0.46 | 0.37 | 0.58 | **0.93** | 0.43 | 0.56 | 0.42 | 0.55 | 0.56 | 0.51 | 0.46 | 0.64 | 0.56 |
| CA=.87 | HT2 | 0.28 | 0.16 | 0.56 | **0.75** | 0.22 | 0.51 | 0.29 | 0.49 | 0.45 | 0.44 | 0.48 | 0.42 | 0.42 |
| CR=0.91 | HT3 | 0.58 | 0.31 | 0.59 | **0.80** | 0.31 | 0.51 | 0.43 | 0.61 | 0.63 | 0.55 | 0.49 | 0.72 | 0.69 |
| AVE=0.73 | HT4 | 0.46 | 0.43 | 0.55 | **0.93** | 0.49 | 0.56 | 0.49 | 0.57 | 0.58 | 0.53 | 0.46 | 0.70 | 0.62 |
| **Facilitation Conditions (FC)** | **FC1** | 0.31 | 0.56 | 0.19 | 0.33 | **0.85** | 0.33 | 0.46 | 0.41 | 0.42 | 0.42 | 0.29 | 0.36 | 0.32 |
| CA=.84 | **FC2** | 0.37 | 0.64 | 0.25 | 0.40 | **0.87** | 0.38 | 0.48 | 0.38 | 0.42 | 0.39 | 0.24 | 0.41 | 0.32 |
| CR=0.89 | **FC3** | 0.38 | 0.59 | 0.28 | 0.41 | **0.91** | 0.43 | 0.53 | 0.43 | 0.44 | 0.46 | 0.30 | 0.36 | 0.31 |
| AVE=0.68 | **FC4** | 0.24 | 0.33 | 0.24 | 0.27 | **0.64** | 0.33 | 0.46 | 0.42 | 0.45 | 0.35 | 0.35 | 0.24 | 0.28 |
| **Hedonic Motivation (HM)** | **HM1** | 0.39 | 0.44 | 0.45 | 0.56 | 0.45 | **0.94** | 0.43 | 0.56 | 0.51 | 0.50 | 0.54 | 0.51 | 0.50 |
| CA=.90 | **HM2** | 0.48 | 0.46 | 0.44 | 0.67 | 0.44 | **0.92** | 0.48 | 0.61 | 0.58 | 0.54 | 0.58 | 0.63 | 0.56 |
| CR=0.94 | **HM3** | 0.25 | 0.26 | 0.38 | 0.44 | 0.30 | **0.87** | 0.37 | 0.51 | 0.44 | 0.43 | 0.54 | 0.43 | 0.42 |
| AVE=0.83 | | | | | | | | | | | | | | |
| **Price Value (PV)** | **PV1** | 0.31 | 0.36 | 0.16 | 0.36 | 0.55 | 0.35 | **0.92** | 0.48 | 0.48 | 0.45 | 0.41 | 0.39 | 0.38 |
| CA=.97 | **PV2** | 0.35 | 0.38 | 0.27 | 0.50 | 0.56 | 0.46 | **0.96** | 0.56 | 0.55 | 0.53 | 0.48 | 0.53 | 0.52 |
| CR=0.98 | **PV3** | 0.32 | 0.39 | 0.29 | 0.48 | 0.53 | 0.51 | **0.92** | 0.54 | 0.54 | 0.52 | 0.44 | 0.47 | 0.46 |
| AVE=0.94 | | | | | | | | | | | | | | |
| **Personal Empowerment** | **PEM-PL1** | 0.54 | 0.41 | 0.58 | 0.61 | 0.45 | 0.49 | 0.53 | 0.84 | **0.91** | 0.73 | 0.67 | 0.58 | 0.63 |
| **Professional Logic (PEM-PL)** | **PEM-PL2** | 0.58 | 0.40 | 0.53 | 0.62 | 0.48 | 0.51 | 0.55 | 0.82 | **0.90** | 0.71 | 0.65 | 0.61 | 0.62 |
| CA=.93 | **PEM-PL3** | 0.56 | 0.39 | 0.55 | 0.56 | 0.44 | 0.52 | 0.48 | 0.87 | **0.92** | 0.75 | 0.73 | 0.57 | 0.61 |
| CR=0.95 | **PEM-PL4** | 0.60 | 0.44 | 0.52 | 0.59 | 0.50 | 0.53 | 0.46 | 0.84 | **0.89** | 0.74 | 0.68 | 0.59 | 0.62 |
| AVE=0.87 | **PEM-PL5** | 0.47 | 0.29 | 0.45 | 0.47 | 0.38 | 0.43 | 0.42 | 0.70 | **0.73** | 0.64 | 0.58 | 0.45 | 0.48 |
| **Personal Empowerment** | **PEM-CL1** | 0.57 | 0.41 | 0.49 | 0.55 | 0.48 | 0.47 | 0.45 | 0.81 | 0.76 | **0.86** | 0.65 | 0.56 | 0.58 |
| **Consumer Logic (PEM-CL)** | **PEM-CL2** | 0.53 | 0.29 | 0.50 | 0.50 | 0.40 | 0.43 | 0.49 | 0.78 | 0.67 | **0.90** | 0.62 | 0.51 | 0.54 |
| CA=.92 | **PEM-CL3** | 0.55 | 0.36 | 0.55 | 0.54 | 0.49 | 0.53 | 0.53 | 0.84 | 0.73 | **0.93** | 0.69 | 0.58 | 0.61 |
| CR=0.94 | **PEM-CL4** | 0.48 | 0.31 | 0.50 | 0.53 | 0.39 | 0.50 | 0.45 | 0.85 | 0.75 | **0.88** | 0.75 | 0.53 | 0.60 |
| AVE=0.76 | | | | | | | | | | | | | | |
| **Personal Empowerment** | **PEM-CCL1** | 0.40 | 0.18 | 0.47 | 0.50 | 0.28 | 0.56 | 0.37 | 0.79 | 0.64 | 0.67 | **0.86** | 0.52 | 0.53 |
| **Community Logic (PEM-CCL)** | **PEM-CCL2** | 0.35 | 0.19 | 0.42 | 0.41 | 0.21 | 0.47 | 0.40 | 0.79 | 0.63 | 0.67 | **0.89** | 0.43 | 0.49 |
| CA=.91 | **PEM-CCL3** | 0.35 | 0.27 | 0.52 | 0.52 | 0.34 | 0.55 | 0.45 | 0.82 | 0.69 | 0.66 | **0.91** | 0.55 | 0.57 |
| CR=0.94 | **PEM-CCL4** | 0.46 | 0.29 | 0.52 | 0.55 | 0.36 | 0.58 | 0.47 | 0.86 | 0.75 | 0.71 | **0.90** | 0.60 | 0.60 |
| AVE=0.80 | **PEM-CCL5** | 0.33 | 0.25 | 0.50 | 0.45 | 0.35 | 0.53 | 0.44 | 0.78 | 0.63 | 0.66 | **0.85** | 0.49 | 0.53 |
| **Behaviour Intention (BI)** | **BI1** | 0.61 | 0.32 | 0.53 | 0.67 | 0.39 | 0.52 | 0.46 | 0.64 | 0.62 | 0.59 | 0.55 | **0.95** | 0.81 |
| CA=.95 | **BI2** | 0.52 | 0.29 | 0.62 | 0.72 | 0.37 | 0.58 | 0.45 | 0.61 | 0.58 | 0.54 | 0.56 | **0.93** | 0.80 |
| CR=0.97 | **BI3** | 0.60 | 0.37 | 0.59 | 0.71 | 0.44 | 0.57 | 0.52 | 0.65 | 0.63 | 0.61 | 0.56 | **0.97** | 0.80 |
| AVE=0.90 | | | | | | | | | | | | | | |
| **Behaviour Intention to** | **BIR1** | 0.56 | 0.23 | 0.61 | 0.64 | 0.29 | 0.50 | 0.45 | 0.65 | 0.62 | 0.61 | 0.58 | 0.78 | **0.93** |
| **Recommend (BIR)** | | | | | | | | | | | | | | |
| CA=.93 | **BIR2** | 0.50 | 0.30 | 0.38 | 0.47 | 0.34 | 0.44 | 0.49 | 0.54 | 0.54 | 0.52 | 0.43 | 0.66 | **0.83** |
| CR=0.95 | **BIR3** | 0.57 | 0.28 | 0.59 | 0.68 | 0.36 | 0.53 | 0.45 | 0.68 | 0.65 | 0.64 | 0.60 | 0.82 | **0.96** |
| AVE=0.83 | **BIR4** | 0.58 | 0.30 | 0.58 | 0.66 | 0.36 | 0.53 | 0.41 | 0.69 | 0.67 | 0.61 | 0.62 | 0.80 | **0.92** |

**Table A.3.2** WCHC Sample Loadings and cross-loadings

| **Constructs** | **Item** | **PE** | **EE** | **SI** | **HT** | **FC** | **HM** | **PV** | **PEM** | **PEMPL** | **PEMCL** | **PEM-CCL** | **BI** | **BIR** |
|---|---|---|---|---|---|---|---|---|---|---|---|---|---|---|
| **Performance Expectancy (PE)** | **PE1** | **0.90** | 0.52 | 0.34 | 0.61 | 0.38 | 0.49 | 0.28 | 0.54 | 0.51 | 0.46 | 0.44 | 0.69 | 0.64 |
| CA=.91 | **PE2** | **0.92** | 0.58 | 0.34 | 0.64 | 0.52 | 0.61 | 0.40 | 0.53 | 0.55 | 0.45 | 0.39 | 0.69 | 0.60 |
| CR=0.94 | **PE3** | **0.94** | 0.54 | 0.34 | 0.59 | 0.45 | 0.51 | 0.41 | 0.56 | 0.58 | 0.48 | 0.39 | 0.74 | 0.64 |
| AVE=0.84 | | | | | | | | | | | | | | |
| **Effort Expectancy (EE)** | **EE1** | 0.54 | **0.93** | 0.27 | 0.60 | 0.68 | 0.59 | 0.42 | 0.36 | 0.32 | 0.33 | 0.29 | 0.46 | 0.47 |
| CA=.94 | **EE2** | 0.60 | **0.94** | 0.40 | 0.64 | 0.71 | 0.60 | 0.52 | 0.39 | 0.37 | 0.32 | 0.33 | 0.51 | 0.49 |
| CR=0.95 | **EE3** | 0.51 | **0.89** | 0.42 | 0.56 | 0.70 | 0.62 | 0.55 | 0.39 | 0.36 | 0.32 | 0.35 | 0.45 | 0.47 |
| AVE=0.84 | **EE4** | 0.54 | **0.91** | 0.22 | 0.59 | 0.64 | 0.56 | 0.45 | 0.40 | 0.37 | 0.34 | 0.32 | 0.54 | 0.44 |
| **Social Influence (SI)** | **SI1** | 0.36 | 0.35 | **0.97** | 0.47 | 0.32 | 0.36 | 0.42 | 0.40 | 0.41 | 0.32 | 0.40 | 0.31 | 0.34 |
| CA=.95 | **SI2** | 0.38 | 0.32 | **0.97** | 0.47 | 0.28 | 0.34 | 0.36 | 0.36 | 0.42 | 0.32 | 0.36 | 0.32 | 0.34 |
| CR=0.97 | **SI3** | 0.34 | 0.36 | **0.95** | 0.47 | 0.26 | 0.34 | 0.37 | 0.37 | 0.48 | 0.34 | 0.37 | 0.35 | 0.39 |
| AVE=0.91 | | | | | | | | | | | | | | |
| **Habit (HT)** | HT1 | 0.62 | 0.49 | 0.42 | **0.89** | 0.49 | 0.61 | 0.42 | 0.49 | 0.49 | 0.42 | 0.36 | 0.64 | 0.55 |
| CA=.87 | HT2 | 0.35 | 0.24 | 0.36 | **0.72** | 0.24 | 0.50 | 0.27 | 0.42 | 0.37 | 0.35 | 0.39 | 0.47 | 0.43 |
| CR=0.91 | HT3 | 0.68 | 0.56 | 0.44 | **0.88** | 0.56 | 0.67 | 0.48 | 0.64 | 0.64 | 0.53 | 0.49 | 0.68 | 0.66 |
| AVE=0.72 | HT4 | 0.58 | 0.54 | 0.43 | **0.90** | 0.54 | 0.65 | 0.42 | 0.58 | 0.55 | 0.49 | 0.48 | 0.65 | 0.59 |
| **Facilitation Conditions (FC)** | **FC1** | 0.39 | 0.63 | 0.28 | 0.47 | **0.87** | 0.53 | 0.54 | 0.40 | 0.40 | 0.34 | 0.30 | 0.41 | 0.46 |
| CA=.90 | **FC2** | 0.44 | 0.82 | 0.24 | 0.52 | **0.89** | 0.58 | 0.53 | 0.38 | 0.35 | 0.34 | 0.32 | 0.46 | 0.44 |
| CR=0.93 | **FC3** | 0.52 | 0.69 | 0.22 | 0.54 | **0.93** | 0.66 | 0.62 | 0.45 | 0.47 | 0.34 | 0.35 | 0.56 | 0.47 |
| AVE=0.77 | **FC4** | 0.36 | 0.48 | 0.30 | 0.39 | **0.82** | 0.54 | 0.64 | 0.38 | 0.41 | 0.29 | 0.28 | 0.50 | 0.33 |
| **Hedonic Motivation (HM)** | **HM1** | 0.59 | 0.63 | 0.29 | 0.70 | 0.63 | **0.96** | 0.55 | 0.58 | 0.56 | 0.49 | 0.48 | 0.60 | 0.56 |
| CA=.91 | **HM2** | 0.61 | 0.69 | 0.40 | 0.75 | 0.70 | **0.94** | 0.63 | 0.58 | 0.58 | 0.47 | 0.44 | 0.59 | 0.53 |
| CR=0.94 | **HM3** | 0.35 | 0.41 | 0.30 | 0.49 | 0.46 | **0.85** | 0.47 | 0.32 | 0.29 | 0.24 | 0.30 | 0.39 | 0.33 |
| AVE=0.84 | | | | | | | | | | | | | | |
| **Price Value (PV)** | **PV1** | 0.32 | 0.44 | 0.34 | 0.38 | 0.61 | 0.55 | **0.92** | 0.45 | 0.43 | 0.44 | 0.32 | 0.40 | 0.37 |
| CA=.96 | **PV2** | 0.36 | 0.48 | 0.35 | 0.42 | 0.63 | 0.52 | **0.95** | 0.41 | 0.42 | 0.37 | 0.28 | 0.46 | 0.39 |
| CR=0.97 | **PV3** | 0.41 | 0.53 | 0.41 | 0.50 | 0.61 | 0.60 | **0.91** | 0.56 | 0.53 | 0.49 | 0.45 | 0.53 | 0.54 |
| AVE=0.93 | | | | | | | | | | | | | | |
| **Personal Empowerment** | **PEM-PL1** | 0.51 | 0.40 | 0.50 | 0.55 | 0.46 | 0.52 | 0.54 | 0.79 | **0.89** | 0.59 | 0.55 | 0.56 | 0.58 |
| **Professional Logic (PEM-PL)** | **PEM-PL2** | 0.49 | 0.36 | 0.46 | 0.60 | 0.42 | 0.54 | 0.47 | 0.83 | **0.92** | 0.64 | 0.60 | 0.53 | 0.58 |
| CA=.92 | **PEM-PL3** | 0.56 | 0.28 | 0.39 | 0.49 | 0.35 | 0.44 | 0.41 | 0.82 | **0.93** | 0.62 | 0.57 | 0.54 | 0.58 |
| CR=0.95 | **PEM-PL4** | 0.57 | 0.37 | 0.35 | 0.56 | 0.42 | 0.46 | 0.42 | 0.76 | **0.89** | 0.53 | 0.53 | 0.66 | 0.62 |
| AVE=0.86 | **PEM-PL5** | 0.46 | 0.27 | 0.26 | 0.43 | 0.39 | 0.39 | 0.33 | 0.62 | **0.69** | 0.49 | 0.41 | 0.49 | 0.45 |
| **Personal Empowerment** | **PEM-CL1** | 0.54 | 0.37 | 0.42 | 0.55 | 0.36 | 0.43 | 0.40 | 0.78 | 0.70 | **0.83** | 0.53 | 0.60 | 0.63 |
| **Consumer Logic (PEM-CL)** | **PEM-CL2** | 0.39 | 0.23 | 0.22 | 0.38 | 0.27 | 0.36 | 0.37 | 0.70 | 0.45 | **0.88** | 0.59 | 0.42 | 0.42 |
| CA=.92 | **PEM-CL3** | 0.44 | 0.32 | 0.33 | 0.44 | 0.27 | 0.38 | 0.43 | 0.79 | 0.58 | **0.92** | 0.64 | 0.47 | 0.55 |
| CR=0.94 | **PEM-CL4** | 0.35 | 0.30 | 0.18 | 0.45 | 0.38 | 0.39 | 0.42 | 0.71 | 0.53 | **0.80** | 0.58 | 0.48 | 0.48 |
| AVE=0.76 | | | | | | | | | | | | | | |
| **Personal Empowerment** | **PEM-CCL1** | 0.34 | 0.36 | 0.36 | 0.42 | 0.30 | 0.36 | 0.36 | 0.76 | 0.52 | 0.59 | **0.88** | 0.45 | 0.48 |
| **Community Logic (PEM-CCL)** | **PEM-CCL2** | 0.41 | 0.34 | 0.30 | 0.45 | 0.36 | 0.42 | 0.37 | 0.77 | 0.53 | 0.57 | **0.91** | 0.48 | 0.50 |
| CA=.88 | **PEM-CCL3** | 0.44 | 0.40 | 0.32 | 0.49 | 0.45 | 0.49 | 0.37 | 0.78 | 0.53 | 0.60 | **0.90** | 0.56 | 0.52 |
| CR=0.92 | **PEM-CCL4** | 0.47 | 0.32 | 0.41 | 0.49 | 0.29 | 0.41 | 0.37 | 0.83 | 0.65 | 0.62 | **0.89** | 0.61 | 0.56 |
| AVE=0.74 | **PEM-CCL5** | 0.28 | 0.12 | 0.32 | 0.35 | 0.16 | 0.30 | 0.20 | 0.70 | 0.45 | 0.61 | **0.79** | 0.36 | 0.37 |
| **Behaviour Intention (BI)** | **BI1** | 0.73 | 0.50 | 0.27 | 0.65 | 0.48 | 0.50 | 0.47 | 0.62 | 0.60 | 0.50 | 0.50 | **0.95** | 0.66 |
| CA=.95 | **BI2** | 0.71 | 0.46 | 0.34 | 0.69 | 0.47 | 0.54 | 0.43 | 0.66 | 0.60 | 0.56 | 0.55 | **0.95** | 0.69 |
| CR=0.97 | **BI3** | 0.76 | 0.57 | 0.35 | 0.72 | 0.63 | 0.64 | 0.54 | 0.68 | 0.62 | 0.59 | 0.56 | **0.96** | 0.75 |
| AVE=0.91 | | | | | | | | | | | | | | |
| **Behaviour Intention to Recommend (BIR)** | **BIR1** | 0.66 | 0.53 | 0.39 | 0.66 | 0.49 | 0.58 | 0.55 | 0.71 | 0.65 | 0.63 | 0.57 | 0.74 | **0.92** |
| CA=.92 | **BIR2** | 0.58 | 0.48 | 0.21 | 0.57 | 0.42 | 0.34 | 0.36 | 0.54 | 0.55 | 0.48 | 0.38 | 0.60 | **0.86** |
| CR=0.94 | **BIR3** | 0.62 | 0.43 | 0.34 | 0.58 | 0.41 | 0.49 | 0.38 | 0.60 | 0.55 | 0.52 | 0.50 | 0.67 | **0.93** |
| AVE=0.80 | **BIR4** | 0.56 | 0.38 | 0.38 | 0.53 | 0.41 | 0.47 | 0.39 | 0.61 | 0.55 | 0.54 | 0.51 | 0.60 | **0.88** |

**Table A.3.3** CHC Patients Sample Descriptive Statistics + Correlations + Square root of AVE's (Diagonal red box)

| | MEAN | SD | PE | EE | SI | HT | FC | HM | PV | PEM | PEM -PL | PEM -CL | PEM -CCL | BI | BIR | AGE | EDUC. | GENDER |
|---|---|---|---|---|---|---|---|---|---|---|---|---|---|---|---|---|---|---|
| **PE** | 5.17 | 1.40 | **0.92** | | | | | | | | | | | | | | | |
| **EE** | 5.25 | 1.41 | 0.60 | **0.92** | | | | | | | | | | | | | | |
| **SI** | 4.33 | 1.57 | 0.37 | 0.36 | **0.96** | | | | | | | | | | | | | |
| **HT** | 4.05 | 1.90 | 0.67 | 0.65 | 0.49 | **0.85** | | | | | | | | | | | | |
| **FC** | 5.14 | 1.56 | 0.49 | 0.75 | 0.30 | 0.55 | **0.88** | | | | | | | | | | | |
| **HM** | 4.69 | 1.58 | 0.58 | 0.65 | 0.36 | 0.72 | 0.66 | **0.92** | | | | | | | | | | |
| **PV** | 4.24 | 1.39 | 0.40 | 0.53 | 0.40 | 0.47 | 0.67 | 0.61 | **0.93** | | | | | | | | | |
| **PEM** | 4.93 | 1.34 | 0.59 | 0.42 | 0.46 | 0.63 | 0.46 | 0.56 | 0.52 | **0.76** | | | | | | | | |
| **PEM-PL** | 4.30 | 1.52 | 0.59 | 0.39 | 0.46 | 0.61 | 0.47 | 0.54 | 0.50 | 0.88 | **0.87** | | | | | | | |
| **PEM-CL** | 4.29 | 1.55 | 0.50 | 0.36 | 0.34 | 0.53 | 0.37 | 0.45 | 0.47 | 0.87 | 0.66 | **0.86** | | | | | | |
| **PEM-CCL** | 4.97 | 1.48 | 0.44 | 0.35 | 0.39 | 0.51 | 0.36 | 0.45 | 0.38 | 0.88 | 0.62 | 0.68 | **0.88** | | | | | |
| **BI** | 5.17 | 1.40 | 0.77 | 0.54 | 0.34 | 0.72 | 0.55 | 0.59 | 0.51 | 0.69 | 0.64 | 0.58 | 0.57 | **0.95** | | | | |
| **BIR** | 5.25 | 1.48 | 0.68 | 0.51 | 0.37 | 0.66 | 0.49 | 0.53 | 0.48 | 0.69 | 0.65 | 0.61 | 0.55 | 0.73 | **0.89** | | | |
| **AGE** | 470.66 | 14.25 | -0.18 | -0.31 | 0.20 | -0.13 | -0.14 | -0.16 | -0.09 | -0.03 | 0.03 | -0.09 | -0.02 | -0.16 | -0.13 | 1.00 | | |
| **EDUC.** | *NA* | *NA* | 0.19 | 0.23 | -0.07 | 0.08 | 0.29 | 0.07 | 0.13 | 0.06 | 0.11 | 0.09 | -0.03 | 0.18 | 0.22 | -0.25 | 1.00 | |
| **GENDER** | *NA* | *NA* | 0.06 | -0.14 | 0.05 | 0.00 | -0.04 | 0.08 | -0.05 | 0.21 | 0.19 | 0.24 | 0.14 | 0.06 | 0.10 | 0.20 | -0.10 | 1.00 |

**Notes:** PE: Performance Expectancy; EE: Effort Expectancy; SI: Social Influence; HT: Habit; FC: Facilitation Conditions; HM: Hedonic Motivation; PV: Price Value; PEM: Personal Empowerment (2nd order);PEM-PL: Personal Empowerment – Professional Logic; PEM-CL: Personal Empowerment- Consumer Logic; PEM-CCL: Personal Empowerment – Community Logic; BI: Behaviour Intention; UB: BIR: Behaviour Intention to Recommend; Educ.: Education

**Table A.3.4** Without CHC Sample Descriptive Statistics + Correlations + Square root of AVE's (Diagonal red box)

| | MEAN | SD | PE | EE | SI | HT | FC | HM | PV | PEM | PEM-PL | PEM-CL | PEM-CCL | BI | BIR | AGE | EDUC. | GENDER |
|---|---|---|---|---|---|---|---|---|---|---|---|---|---|---|---|---|---|---|
| **PE** | 5.27 | 1.34 | **0.91** | | | | | | | | | | | | | | | |
| **EE** | 5.51 | 1.29 | 0.42 | **0.90** | | | | | | | | | | | | | | |
| **SI** | 3.85 | 1.63 | 0.49 | 0.14 | **0.97** | | | | | | | | | | | | | |
| **HT** | 3.71 | 1.88 | 0.53 | 0.38 | 0.66 | **0.85** | | | | | | | | | | | | |
| **FC** | 5.20 | 1.54 | 0.40 | 0.66 | 0.29 | 0.43 | **0.82** | | | | | | | | | | | |
| **HM** | 4.37 | 1.57 | 0.43 | 0.44 | 0.47 | 0.63 | 0.45 | **0.91** | | | | | | | | | | |
| **PV** | 4.41 | 1.40 | 0.35 | 0.40 | 0.26 | 0.48 | 0.58 | 0.48 | **0.93** | | | | | | | | | |
| **PEM** | 4.44 | 1.56 | 0.60 | 0.40 | 0.62 | 0.65 | 0.49 | 0.62 | 0.57 | **0.81** | | | | | | | | |
| **PEM-PL** | 4.64 | 1.48 | 0.43 | 0.45 | 0.60 | 0.65 | 0.52 | 0.57 | 0.56 | 0.88 | **0.87** | | | | | | | |
| **PEM-CL** | 4.53 | 1.54 | 0.60 | 0.38 | 0.57 | 0.59 | 0.49 | 0.54 | 0.54 | 0.87 | 0.82 | **0.89** | | | | | | |
| **PEM-CCL** | 4.17 | 1.58 | 0.63 | 0.27 | 0.55 | 0.55 | 0.35 | 0.61 | 0.48 | 0.88 | 0.76 | 0.76 | **0.88** | | | | | |
| **BI** | 4.86 | 1.71 | 0.61 | 0.35 | 0.61 | 0.74 | 0.42 | 0.59 | 0.50 | 0.69 | 0.64 | 0.61 | 0.59 | **0.95** | | | | |
| **BIR** | 4.84 | 1.71 | 0.61 | 0.31 | 0.60 | 0.68 | 0.37 | 0.55 | 0.49 | 0.69 | 0.68 | 0.65 | 0.62 | 0.85 | **0.91** | | | |
| **AGE** | 35.26 | 1.86 | 0.03 | -0.25 | 0.19 | 0.10 | -0.06 | 0.09 | -0.09 | 0.06 | 0.06 | 0.04 | 0.05 | 0.06 | 0.04 | 1.00 | | |
| **EDUCATION** | *NA* | *NA* | -0.07 | 0.14 | -0.08 | -0.07 | 0.22 | 0.13 | 0.08 | 0.01 | 0.02 | 0.02 | -0.02 | -0.02 | 0.01 | 0.18 | 1.00 | |
| **GENDER** | *NA* | *NA* | 0.29 | 0.15 | 0.22 | 0.19 | 0.19 | 0.17 | 0.12 | 0.22 | 0.25 | 0.19 | 0.17 | 0.24 | 0.22 | 0.13 | 0.04 | 1.00 |

**Notes:** PE: Performance Expectancy; EE: Effort Expectancy; SI: Social Influence; HT: Habit; FC: Facilitation Conditions; HM: Hedonic Motivation; PV: Price Value; PEM: Personal Empowerment (2nd order);PEM-PL: Personal Empowerment – Professional Logic; PEM-CL: Personal Empowerment- Consumer Logic; PEM-CCL: Personal Empowerment – Community Logic; BI: Behaviour Intention; UB: BIR: Behaviour Intention to Recommend; Educ.: Education

**Table A.3.5** CHC Patients Sample Formative Indicators Quality Criteria

| Indicator | VIF | Loading | Weight |
|---|---|---|---|
| **UB1** | 2.08 | 0.74** | 0.31* |
| **UB2** | 1.70 | 0.39** | 0.08** |
| **UB3** | 1.93 | 0.85** | 0.56ns |
| **UB4** | 2.39 | 0.77** | 0.20ns |
| **UB5** | 2.26 | 0.57** | -0.18 ns |
| **UB6** | 1.89 | 0.43** | 0.04* |
| **UB7** | 2.67 | 0.69** | 0.40 ns |
| **UB8** | 2.38 | 0.52ns | -0.13 ns |
| **UB9** | 1.83 | 0.26ns | 0.03 ns |
| **UB10** | 1.70 | 0.39** | 0.08 ns |

**Notes:** (** *P* <.01; * *P* < .05; ns= non-significant)

**Table A.3.6** Without CHC Sample Formative Indicators Quality Criteria

| Indicator | VIF | Loading | Weight |
|---|---|---|---|
| **UB1** | 2.24 | 0.73** | 0.16ns |
| **UB2** | 2.03 | 0.47** | 0.02** |
| **UB3** | 1.89 | 0.83** | 0.43** |
| **UB4** | 1.94 | 0.79** | 0.37ns |
| **UB5** | 1.97 | 0.51** | -0.04ns |
| **UB6** | 1.94 | 0.53** | 0.09ns |
| **UB7** | 1.67 | 0.60** | 0.05** |
| **UB8** | 2.10 | 0.67** | 0.26ns |
| **UB9** | 2.04 | 0.45** | 0.08ns |
| **UB10** | 2.03 | 0.47** | 0.02ns |

**Notes:** (** *P* <.01; * *P* < .05; ns= non-significant)

**Table A.3.7** CHC Patients Sample Formative Indicators Quality Criteria

| Higher-Order Formative Construct | First-order Reflective Constructs | VIF | Weight |
|---|---|---|---|
| Personal Empowerment (PEM) | Personal Empowerment - Community Logic (PEM-CCL) | 2.05 | 0.40** |
| | Personal Empowerment - Consumer Logic (PEM-CL) | 2.27 | 0.31** |
| | Personal Empowerment - Professional Logic (PEM-PL) | 1.95 | 0.43** |

**Notes:** (** *P* <.01; * *P* < .05; ns= non-significant)

**Table A.3.8** Without CHC Sample Formative Indicators Quality Criteria

| Higher-Order Formative Construct | First-order Reflective Constructs | VIF | Weight |
|---|---|---|---|
| Personal Empowerment (PEM) | Personal Empowerment - Community Logic (PEM-CCL) | 2.75 | 0.37** |
| | Personal Empowerment - Consumer Logic (PEM-CL) | 3.54 | 0.32** |

| | | | |
|---|---|---|---|
| | Personal Empowerment - Professional Logic (PEM-PL) | 3.51 | 0.39** |

**Notes:** (** *P* <.01; * *P* < .05; ns= non-significant)

**Table A.3.9** CHCP Sample Heterotrait-Monotrait Ratio (HTMT)

| | PE | EE | SI | HT | FC | HM | PV | PEM | PEM -PL | PEM -CL | PEM -CCL | BI | BIR | | AGE | EDUC. | GENDER |
|---|---|---|---|---|---|---|---|---|---|---|---|---|---|---|---|---|---|
| **PE** | | | | | | | | | | | | | | | | | |
| **EE** | 0.65 | | | | | | | | | | | | | | | | |
| **SI** | 0.40 | 0.38 | | | | | | | | | | | | | | | |
| **HT** | 0.74 | 0.70 | 0.53 | | | | | | | | | | | | | | |
| **FC** | 0.54 | 0.81 | 0.32 | 0.60 | | | | | | | | | | | | | |
| **HM** | 0.62 | 0.68 | 0.39 | 0.79 | 0.72 | | | | | | | | | | | | |
| **PV** | 0.43 | 0.57 | 0.42 | 0.51 | 0.73 | 0.65 | | | | | | | | | | | |
| **PEM** | 0.63 | 0.44 | 0.48 | 0.69 | 0.50 | 0.58 | 0.55 | | | | | | | | | | |
| **PEM-PL** | 0.65 | 0.42 | 0.48 | 0.67 | 0.52 | 0.57 | 0.54 | 0.94 | | | | | | | | | |
| **PEM-CL** | 0.56 | 0.39 | 0.36 | 0.60 | 0.42 | 0.49 | 0.52 | 0.96 | 0.66 | | | | | | | | |
| **PEM-CCL** | 0.48 | 0.38 | 0.41 | 0.56 | 0.39 | 0.48 | 0.41 | 0.94 | 0.73 | 0.76 | | | | | | | |
| **BI** | 0.83 | 0.56 | 0.35 | 0.79 | 0.59 | 0.62 | 0.53 | 0.72 | 0.69 | 0.63 | 0.60 | | | | | | |
| **BIR** | 0.75 | 0.55 | 0.39 | 0.73 | 0.53 | 0.56 | 0.50 | 0.73 | 0.70 | 0.67 | 0.59 | 0.78 | | | | | |
| **AGE** | 0.19 | 0.32 | 0.20 | 0.14 | 0.15 | 0.16 | 0.10 | 0.08 | 0.06 | 0.10 | 0.07 | 0.16 | 0.13 | | | | |
| **EDUC.** | 0.20 | 0.23 | 0.07 | 0.11 | 0.31 | 0.10 | 0.14 | 0.12 | 0.12 | 0.09 | 0.11 | 0.18 | 0.23 | | 0.25 | | |
| **GENDER** | 0.06 | 0.15 | 0.05 | 0.07 | 0.04 | 0.09 | 0.05 | 0.22 | 0.20 | 0.25 | 0.15 | 0.06 | 0.10 | | 0.20 | 0.10 | |

**Notes:** PE: Performance Expectancy; EE: Effort Expectancy; SI: Social Influence; HT: Habit; FC: Facilitation Conditions; HM: Hedonic Motivation; PV: Price Value; PEM: Personal Empowerment (2nd order);PEM-PL: Personal Empowerment – Professional Logic; PEM-CL: Personal Empowerment- Consumer Logic; PEM-CCL: Personal Empowerment – Community Logic; BI: Behaviour Intention; UB: Use behaviour BIR: Behaviour Intention to Recommend; Educ.: Education;

**Table A.3.10** WCHC Sample Heterotrait-Monotrait Ratio (HTMT)

| | PE | EE | SI | HT | FC | HM | PV | PEM | PEM -PL | PEM -CL | PEM -CCL | BI | BIR | | AGE | EDUC. | GENDER |
|---|---|---|---|---|---|---|---|---|---|---|---|---|---|---|---|---|---|
| **PE** | | | | | | | | | | | | | | | | | |
| **EE** | 0.45 | | | | | | | | | | | | | | | | |
| **SI** | 0.54 | 0.14 | | | | | | | | | | | | | | | |
| **HT** | 0.59 | 0.41 | 0.73 | | | | | | | | | | | | | | |
| **FC** | 0.45 | 0.74 | 0.33 | 0.49 | | | | | | | | | | | | | |
| **HM** | 0.45 | 0.46 | 0.50 | 0.69 | 0.51 | | | | | | | | | | | | |
| **PV** | 0.37 | 0.44 | 0.27 | 0.52 | 0.67 | 0.51 | | | | | | | | | | | |
| **PEM** | 0.64 | 0.42 | 0.65 | 0.71 | 0.55 | 0.67 | 0.60 | | | | | | | | | | |
| **PEM-PL** | 0.69 | 0.48 | 0.64 | 0.73 | 0.60 | 0.62 | 0.60 | 0.99 | | | | | | | | | |
| **PEM-CL** | 0.66 | 0.42 | 0.61 | 0.66 | 0.57 | 0.59 | 0.58 | 0.98 | 0.89 | | | | | | | | |
| **PEM-CCL** | 0.47 | 0.28 | 0.58 | 0.62 | 0.41 | 0.66 | 0.52 | 0.97 | 0.82 | 0.82 | | | | | | | |
| **BI** | 0.65 | 0.36 | 0.63 | 0.80 | 0.47 | 0.62 | 0.53 | 0.70 | 0.69 | 0.66 | 0.63 | | | | | | |
| **BIR** | 0.66 | 0.33 | 0.63 | 0.74 | 0.43 | 0.59 | 0.53 | 0.74 | 0.73 | 0.71 | 0.66 | 0.90 | | | | | |
| **AGE** | 0.07 | 0.26 | 0.19 | 0.11 | 0.06 | 0.09 | 0.09 | 0.07 | 0.06 | 0.06 | 0.06 | 0.06 | 0.04 | | | | |
| **EDUC.** | 0.08 | 0.24 | 0.08 | 0.08 | 0.24 | 0.15 | 0.08 | 0.04 | 0.02 | 0.03 | 0.06 | 0.02 | 0.03 | | 0.18 | | |
| **GENDER** | 0.31 | 0.15 | 0.22 | 0.21 | 0.21 | 0.17 | 0.12 | 0.23 | 0.26 | 0.20 | 0.18 | 0.24 | 0.23 | | 0.13 | 0.04 | |

**Notes:** PE: Performance Expectancy; EE: Effort Expectancy; SI: Social Influence; HT: Habit; FC: Facilitation Conditions; HM: Hedonic Motivation; PV: Price Value; PEM: Personal Empowerment (2nd order);PEM-PL: Personal Empowerment – Professional Logic; PEM-CL: Personal Empowerment- Consumer Logic; PEM-CCL: Personal Empowerment – Community Logic; BI: Behaviour Intention; UB: BIR: Behaviour Intention to Recommend; Educ.: Education;
